# Synthetic miRNAs induce dual arboviral-resistance phenotypes in the vector mosquito *Aedes aegypti*

**DOI:** 10.1038/s42003-017-0011-5

**Published:** 2018-02-08

**Authors:** Pei-Shi Yen, Anthony James, Jian-Chiuan Li, Chun-Hong Chen, Anna-Bella Failloux

**Affiliations:** 1 Institut Pasteur, Department of Virology, Unit of Arboviruses and Insect Vectors, Paris, 75015 France; 2 Departments of Microbiology & Molecular Genetics and Molecular Biology & Biochemistry, University of California, Irvine, CA 92697 USA; 3 National Institute of Infectious Diseases and Vaccinology, National Health Research Institutes, Zhunan, Miaoli, 35053 Taiwan

## Abstract

Mosquito-borne arboviruses are responsible for recent dengue, chikungunya, and Zika pandemics. The yellow-fever mosquito, *Aedes aegypti*, plays an important role in the transmission of all three viruses. We developed a miRNA-based approach that results in a dual resistance phenotype in mosquitoes to dengue serotype 3 (DENV-3) and chikungunya (CHIKV) viruses. The target viruses are from two distinct arboviral families and the antiviral mechanism is designed to function through the endogenous miRNA pathway in infected mosquitoes. Challenge experiments showed reductions in viral transmission efficiency of transgenic mosquitoes. Several components of mosquito fitness were examined, and transgenic mosquitoes with the *PUb* promoter showed minor fitness costs at all developing stages. Further development of these strains with gene editing tools could make them candidates for releases in population replacement strategies for sustainable control of multiple arbovirus diseases.

## Abstract (web summary)

Pei-Shi Yen *et al*. report a strategy for engineering transgenic *Aedes aegypti* mosquitoes to express antiviral miRNAs that simultaneously target two distinct arboviruses, dengue and chikungunya. The strategy results in reduced viral transmission in the mosquito saliva following an infected bloodmeal.

## Introduction

Dengue and chikungunya are two major arboviral diseases that have emerged as global threats in the past decades. Approximately 390 million people are infected annually with dengue and over 50% of the world’s population live under the risk of infection, draining annually an estimated $40 billion for health-care spending and lost productivity in affected countries^1^. Compared to dengue, chikungunya has a lesser impact on public health and had been a neglected tropical disease until the 2005 outbreak in La Réunion Island, when one-third of the population was affected. Since then, there have been several chikungunya outbreaks worldwide including in Southeast and East Asia, Central Africa, South Pacific Islands, and lately in Latin America and the Caribbean^2^. Many imported cases have been reported in Europe and North America raising the risk of local transmission. Autochthonous cases of dengue were recorded in Croatia^3^, France^4,5^, and Madeira^6^, while chikungunya has appeared in Italy^7^ and France^8,9^.

Dengue virus (DENV) and chikungunya virus (CHIKV) co-circulate in several tropical areas and co-infections in human are frequently reported^10–22^. The viruses belong to two distinct families but share the same mosquito vectors, *Aedes* species. Mosquitoes can acquire DENV and CHIKV simultaneously after feeding on a co-infected patient or after two consecutive blood meals on viremic hosts^23^. Co-infected mosquitoes can transmit concomitantly DENV and CHIKV to subsequent hosts^24^, and this is likely to cause more severe symptoms than mono-infections^10,25^.

DENV-3 is the fastest spreading DENV serotype in the past two decades^26^. Because a licensed tetravalent dengue vaccine is still not available^27,28^, novel vector control strategies are needed to prevent virus transmission between mosquitoes and hosts. Furthermore, while vaccination would greatly reduce urban transmission, enzootic circulation of arboviruses carries the risk of mutation accumulation and spillover infections that would not be impeded^29–31^. Eliminating both CHIKV and DENV-3 viruses in mosquito vectors would reduce the burden on population health, particularly for countries already under stress in their health-care system.

While most arboviruses can induce significant morbidity and/or mortality in some vertebrate hosts, infections of mosquito vectors are generally considered non-pathogenic^32^. However, interactions between the replicating virus and the mosquito immune defense system may influence subsequent viral dissemination and transmission. Considerable progress has been achieved in understanding the innate defenses of the mosquito against arboviruses. Among them, RNA interference (RNAi) has been shown to be a major innate response of mosquitoes against arboviruses. Knock-down experiments targeting RNAi components such as Dcr2, R2D2, and Ago2 in *Aedes* show increased viral loads or decreased extrinsic incubation periods in mosquitoes^33^. Furthermore, virus replication is suppressible in cultured mosquito cell lines expressing long double-stranded RNA (dsRNA) molecules designed to target the viral genome^34^. RNAi-based, virus-resistant mosquitoes were developed in which transgenes comprising long dsRNAs targeting DENV-2 under the control of a blood meal-inducible gene promoter were able to confer a strong serotype-specific, virus- resistance phenotype^35–37^. According to the species-conserved miRNA processing pathway, the miRNA precursors (pri-miRNA) are processed into ~70 nt hairpins by Drosha in the nucleus, which is followed by exporting into the cytoplasm by Exportin5. In the cytoplasm, the hairpins are cleaved into ~22 nt miRNA duplexes by Dicer-1, which are then loaded into Ago-1 or Ago-2 proteins in miRNA-induced silencing complexes (miRISCs) according to their different structure properties^38–40^. By recognizing the complementary sequence of the target RNA, miRISCs execute silencing through RNA degradation, translational inhibition or both^41,42^.

Here we report the first miRNA-based genetically engineered mosquitoes, to our knowledge, that are refractory to DENV-3 and CHIKV simultaneously. In addition, we show some fitness costs resulting from the transgenes, but anticipate that could be mitigated with additional modifications to the transgenes and their insertion sites.

## Results

### Constructing the artificial antiviral miRNA

**Fig. 1:**
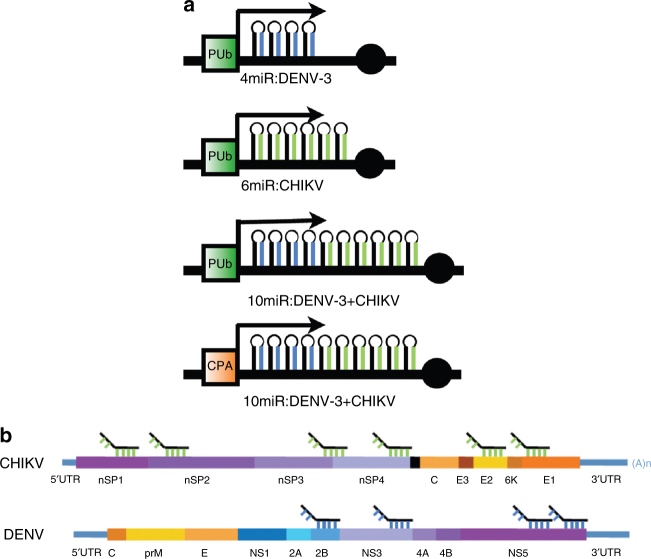
Schematic of the artificial antiviral miRNA. **a** Target genes. **b** Expression cassettes. Based on *mariner* transposon system, the ubiquitous and midgut-specific induction promoters were used for expressing the downstream synthetic miRNAs. AeCPA/PUb promoter, *Ae. aegypti* carboxypeptidase A/PolyUbiquitin promoter; 4miR:DENV-3, anti DENV-3 miRNA cluster for four anti-DENV-3 miRNAs; 6miR:CHIKV, anti CHIKV miRNA cluster for six anti-CHIKV miRNAs; 10miR:DENV-3+CHIKV, anti DENV-3/CHIKV miRNA cluster for four for DENV-3 and six for CHIKV

Two consensus sequences of DENV-3 and CHIKV were defined from 356 and 32 isolates, respectively, of each virus (Supplementary Data 1). Four regions from DENV-3 and six from CHIKV were selected as the targets of antiviral miRNAs on the basis of their sequence coverage and targeted regions (Fig. 1). Corresponding miRNAs were designed and cloned in tandem to make compound anti-viral effector genes. The sequence coverages of the anti-DENV-3 miRNAs to the viruses used to generate the consensus sequence range from 96.6% to 98.6%, and the targeted genes encode the non-structural proteins, NS2B, NS3, and NS5. The anti-CHIKV miRNAs have 96.9–100% identity to the viruses used to generate the consensus sequence, and the targeted genes encode the non-structural proteins, NSP1, NSP2, NSP3-4, NSP4, and the structural proteins E2, and E1 (Supplementary Table 1). In addition, to verify if any miRNA off-target effect might be caused by the synthetic antiviral miRNAs, all the sequences of each antiviral miRNA were examined by the miRNA off-target effect prediction software Genome-wide Enrichment of Seed Sequence matches (GESS)^43^, including the passenger strands of each antiviral miRNA; no statistically significant interaction was identified against any transcript of *Ae. aegypti*. All antiviral miRNA clusters were constructed to place them under either the *Aedes PolyUbiquitin* (AePUB) or *Aedes Carboxypeptidase A* (AeCPA) gene promoters to elicit constitutive or blood meal-inducible, midgut-specific expression of the effector molecules (Fig. 1).

### Generation of transgenic mosquitoes

A Class II TE *mariner MosI* system^44^ was used to generate transgenic mosquito lines by microinjection in four separate experiments mixtures of the donor plasmids, pMosI_AePUb>4miR:D3 (4miR:D3), pMosI_AePUb>6miR:CHIKV (6miR:Chik), pMosI_AePUb>10miR:D3+CHIKV (AePUb>10miR), and pMosI_AeCPA>10miR:D3+CHIKV (AeCPA>10miR), with the transposase expressing helper plasmid pKhsp82MOS. A total of 432, 595, 310, and 355 embryos were injected with each donor plasmid, and of these, 151, 153, 141, and 62, developed into adults. Following outcrossing of G_1_ adults, a total of 1, 5, 3, and 5 lines, respectively, were obtained from each crossing family (Supplementary Table 2). Homozygous lines were generated by screening inter-crossed families in which progeny were 100% reporter-positive for two generations. The copy number of transgenic cassettes in mosquito chromosomes was confirmed by Southern blot analysis using restriction enzymes that have no or only a single cutting site within the transgene and ^32^P-labeled probes complementary to the 10 miRNA cluster region. The results indicate that both mosquito lines contain only a single copy of the transgene cassette in a different locus in the genome (Supplementary Figure 1).

### Expression of artificial miRNAs

**Fig. 2:**
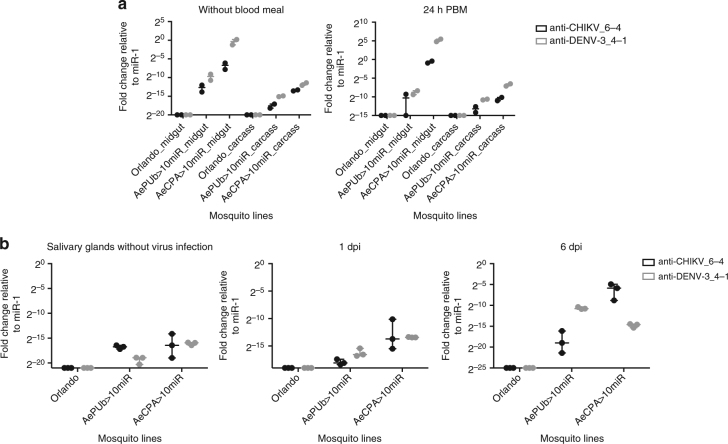
Detection of artificial antiviral miRNAs. **a** In midgut and carcass. **b** In salivary glands. Total RNA were isolated from mosquito midguts and carcasses dissected at 0 and 24 h post blood meal, whereas the RNA of salivary glands were extracted from the mosquitoes co-challenged with CHIKV and DENV-3 at 0, 1, and 6 days after infection. Reverse transcription and qPCR were conducted as described in materials and methods. anti-CHIKV_6-4, the fourth anti-CHIKV miRNA; anti-DENV-3_4-1, the first anti-DENV-3 miRNA. Data were normalized to normalized values of aae-miR-1, and presented in relative expression levels to aae-miR-1. Each sample corresponds to two replicates (2 × 12 mosquitoes)

Signals confirming the expression of anti-CHIKV-4 and anti-DENV3-1 were detected by miRNA qPCR analyses of female midguts and carcasses prepared from tissues collected 0 and 24 h post blood meal (PBM; Fig. 2a, Supplementary Figure 2). The mature miRNAs were polyadenylated, followed by reverse transcription with poly(A)-adapter primer for synthesizing an adapter-linked miRNA complementary DNA (miRNA cDNA). With the miRNA-specific and adapter primers (Supplementary Table 3), the mature miRNA can be detected by qPCR analysis. The antiviral miRNAs of AePUb>10miR mosquitoes were detectable in the midgut and carcass, and a slightly increased expression level was observed 24 h PBM. As the AeCPA promoter is reported to be active in the midgut and salivary glands^45^, the antiviral miRNAs was detected in the midgut and carcass of AeCPA>10miR mosquitoes, and the expression levels were increased 24 h PBM. The two antiviral miRNAs were also detected from the samples of female salivary glands at day 0, 1, and 6 after receiving a viremic blood meal; the results show that both antiviral miRNAs remain detectable in the salivary glands even at day 6 after virus challenge (Fig. 2b, Supplementary Figure 2). We interpret these data to indicate that the expression of the antiviral miRNAs in the midgut, carcass and salivary glands, remains inducible after receiving a blood meal.

### Impacts of transgene on life-table parameters

A number of life-table parameters that might be expected to affect fitness were evaluated. These include larval development time, larval/pupal mortality, adult lifespan, sex ratio, and male mating competitiveness (Table 1).

**Table 1:** Life-table parameters of transgenic mosquitoes

Mosquito lines	Larval development time (days)		Larval mortality rate (%)	Pupal mortality rate (%)	Adult lifespan (days)		Sex ratio (%)	Male mating competitiveness (%)
	Male	Female			Male	Female		
Orlando	6.43 ± 0.03 (307)	7.02±0.05 (265)	1.88 ± 0.56 (583)	2.83 ± 0.67 (600)	39.51 ± 11.58 (150)	44.32± 14.23 (150)	46.32±2.08 (572)	ND
AePUb > 10miR	6.12 ± 0.03 (289)	6.29± 0.03 (254)	4.40 ± 0.86 (568)	2.0 ± 0.57 (600)	36.9 ± 11.75 (150)	41.87 ± 14.91 (150)	46.77 ± 2.14 (543)	58.5 ± 15.8 (41)
AeCPA > 10miR	6.7 ± 0.04 (170)	6.87± 0.05 (115)	16.90 ± 2.02 (343)	7.75 ± 1.33 (400)	23.61 ± 10.0 (150)	28.15 ± 12.83 (150)	40.35 ± 2.91 (285)	26.3 ± 14.7 (38)

Mosquito larval development time, larval/pupal mortality, adult lifespan analysis, sex ratio, and test of male mating competitiveness were conducted at 28 °C. Larval developmental time was determined by the period from the first instar larva to pupal stage; Larval mortality corresponds to the number of emerged adults among analyzed larvae; Pupal mortality corresponds to the number of pupae among emerged adults; Adult life spans were recorded daily by counting the number of dead mosquitoes and separated by sex; Proportion of females was determined by the number of females among all adults; Male mating competitiveness was defined as the proportion of reporter positive individuals compared to negative individuals in the same experimental cage. In brackets, the number of mosquitoes tested is given

*ND* not determined

**Fig. 3:**
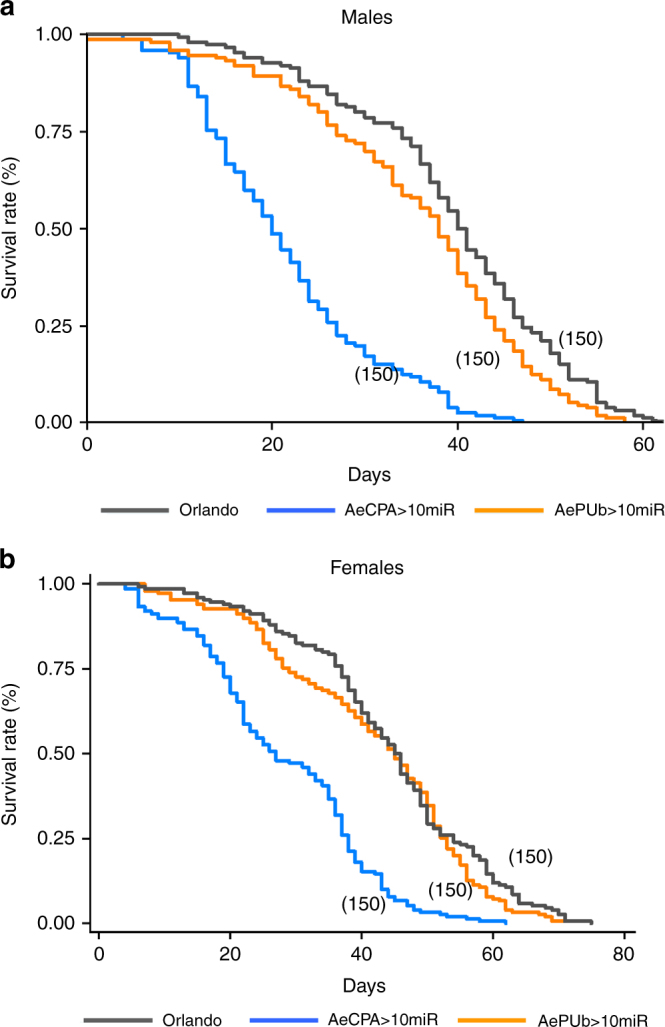
Survival of transgenic mosquitoes. **a** Males. **b** Females. Survival curves were compared between AePUb > 10miR, AeCPA > 10miR and wild-type Orlando mosquitoes. In brackets, the number of mosquitoes is given

In our rearing conditions, wild-type (Orlando) mosquitoes needed an average of 6.43 ± 0.03 (males) and 7.02 ± 0.05 (females) days for development from first instar larvae to pupae, while AePUb>10miR mosquitoes had development times of 6.12 ± 0.03 (males) and 6.29 ± 0.03 (females) days, and AeCPA>10miR had 6.7 ± 0.04 and 6.87 ± 0.05 days for males and females, respectively (Kruskal–Wallis test: *p* < 10^−4^ (males), *p* < 10^−4^ (females)). The larval mortality rate was 4.40 ± 0.86% and 16.90 ± 2.02% for AePUb> 10miR and AeCPA>10miR mosquitoes, respectively, and these latter were significantly higher than wild-type Orlando mosquitoes at 1.88 ± 0.56% (Fisher’s exact test: *p* < 10^−4^). As for the pupal mortality, AeCPA>10miR mosquitoes had a significantly higher pupal mortality rate (Fisher’s exact test: *p* < 10^−4^), 7.75%, compared to AePUb>10miR and wild-type Orlando, 2.0% and 2.83%, respectively. The adult life spans were also analyzed. The mean survival times of AePUb>10miR male and female adults were 36.9 ± 11.75 and 41.78 ± 14.91 days, respectively, which are not significantly different than 39.51 ± 11.58 and 44.32 ± 14.23 days for wild-type Orlando mosquitoes (Kruskal–Wallis test: *p* = 0.07). However, AeCPA > 10miR mosquitoes had shorter survival times with means of 23.61 ± 10.00 and 28.15 ± 12.83 days for males and females, respectively, significantly shorter than wild-type Orlando mosquitoes (Kruskal–Wallis test: *p* < 10^−4^). AeCPA>10miR mosquitoes have a significantly lower survival rate than the two other strains (log rank test: *p* < 0.05) (Fig. 3a and b). Among these adult mosquitoes, the percent of female AeCPA>10miR mosquitoes is 40.35 ± 2.91%, which is lower than 46.32 ± 2.08 % of wild-type Orlando and 46.77 ± 2.14% of AePUb>10miR mosquitoes, there is no significant difference among the three lines (Fisher’s exact test: *p *= 0.17). We conclude that the high larval and pupal mortality rate of AeCPA>10miR mosquitoes is not sex-dependent. Male mating competitiveness of both transgenic lines is determined by mating competition with the same number of wild-type males. Our results show that the mating competitiveness of the AePUb>10miR male was 58.5 ± 7.8%, indicating an advantage when compared with wild-type mosquitoes. For AeCPA>10miR mosquitoes, the proportion of reporter-positive mosquitoes was 26.3 ± 7.2%, supporting the conclusion that they are less competitive in the presence of wild-type mosquitoes. Mating competitiveness of the AePUb>10miR males was significantly higher compared to AeCPA>10miR males in the presence of wild-type males (Fisher’s exact test: *p* = 0.004).

### Virus suppression test of transgenic mosquitoes

**Fig. 4:**
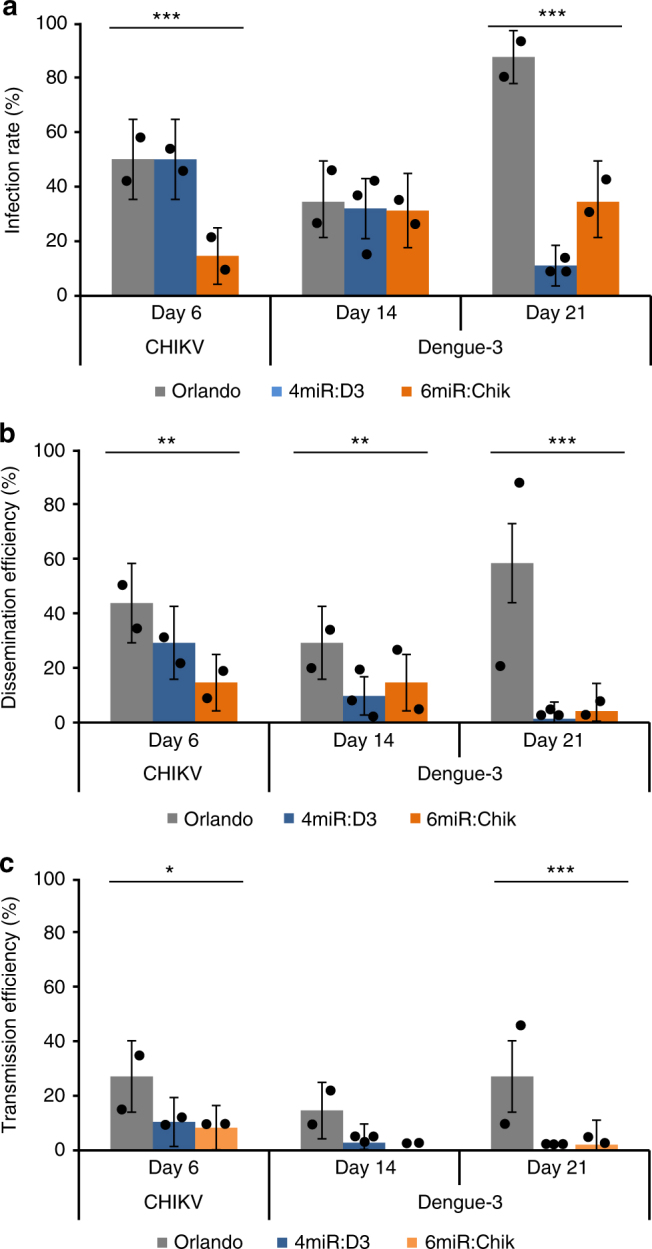
Anti-DENV-3/CHIKV phenotype of transgenic 4miR:D3 and 6miR:Chik mosquitoes. **a** Infection rate. **b** Dissemination rate. **c** Transmission efficiency. Mosquitoes were co-challenged with DENV-3 Cambodia and CHIKV 0621 strain at titer 10^7^ and 10^6^ ffu/mL, respectively. Samples were collected and titrated at 6 and 21 dpi on C6/36 cells. The infection rate was defined as number of positive midgut samples of the total number tested; dissemination efficiency was defined as number of positive head samples of the total number tested; transmission efficiency defined as number of positive saliva among number of tested. Saliva samples were collected via salivation by inserting the proboscis of leg- and wing-less mosquito into a P20 tip containing 5 microliter of FBS, then expelled into 45 microliter of L-15 media after 30 min for analysis. Each sample corresponds to two replicates (2 × 24 mosquitoes) or 3 replicates (3 × 24 mosquitoes). The error bars correspond to the confidence intervals (95%). Significant *p* values are indicated by an asterisk: *p < 0.05, **p < 0.01, ***p < 0.001

The antiviral efficiency of artificial miRNAs for each virus was confirmed separately for AePUb > 4miR:DENV3 and AePUb > 6miR:CHIKV mosquitoes (Fig. 4, Supplementary Figure 3). These mosquitoes were less capable of transmitting CHIKV at 6 days post-infection (dpi) (Orlando (mean ± SE): 27.08 ± 6.4, AePUb > 4miR:DENV3: 10.41 ± 4.45, AePUb > 6miR:CHIKV: 8.33 ± 4.03) and DENV-3 at 21 dpi (Orlando: 27.08 ± 6.48, AePUb > 4miR:DENV3: 0, AePUb > 6miR:CHIKV: 2.08 ± 2.06).

**Fig. 5:**
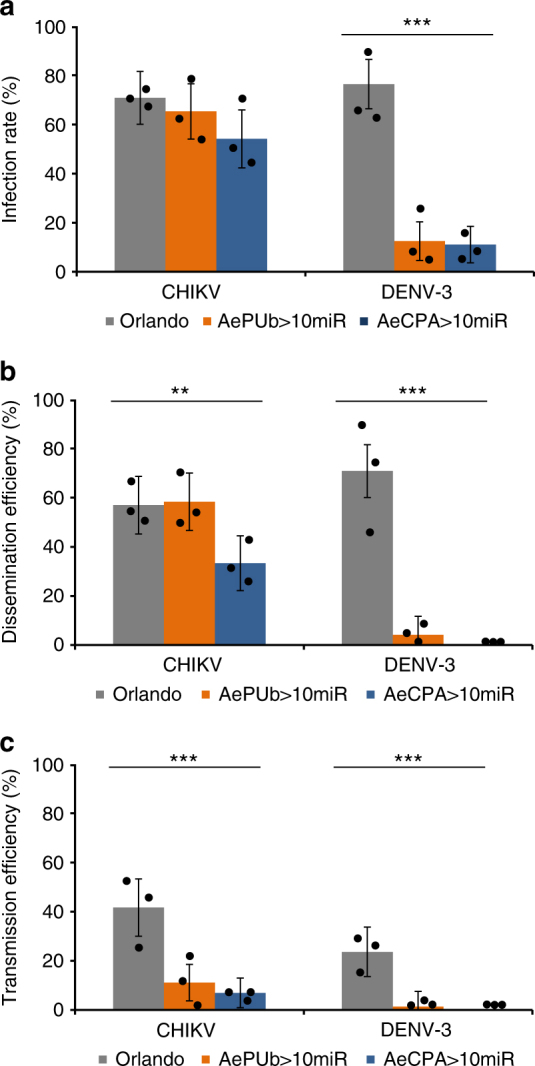
Anti-DENV-3/CHIKV phenotype of transgenic AePUb>10miR and AeCPA>10miR mosquitoes. **a** Infection rate. **b** Dissemination rate. **c** Transmission efficiency. Mosquitoes were co-challenged with DENV-3 Cambodia (Supporting information) and CHIKV 06.21^71^ strains at titers of 10^7^ and 10^6^ ffu/mL, respectively. Samples were collected and titrated at 6 and 21 dpi on C6/36 cells. Infection rate was defined as number of positive body samples among tested ones; dissemination efficiency refers to the number of positive head samples (i.e., successful viral dissemination after passing the midgut barrier) among tested ones; transmission efficiency was defined as the number of positive saliva (i.e., successful transmission) among tested ones. Saliva samples were collected after 30 min in a P20 tip containing 5 µL of FBS and then expelled into 45 µl of L-15 media for analysis. Each sample corresponds to 3 replicates (3 × 24 mosquitoes). The error bars correspond to the confidence intervals (95%). Significant *p* values are indicated by an asterisk: **p < 0.01, ***p < 0.001

We then co-challenged two selected strains from AePUb > 10miR and AeCPA > 10miR mosquito lines with DENV-3 at 10^7^ ffu/mL and CHIKV at 10^6^ ffu/mL. Whole bodies, heads, and saliva were collected for analyzing viral titers. Among three groups of saliva collected from 24 AePUb>10miR and AeCPA>10miR mosquitoes, the CHIKV transmission efficiency was an average of 11.11% and 6.94%, respectively, at 6 dpi, whereas the wild-type Orlando mosquitoes showed an average transmission efficiency of 41.67% (Fig. 5, Supplementary Figure 4).

CHIKV infection and dissemination barriers were assayed by recovering virus particles from bodies and heads, and both AePUb > 10miR and AeCPA > 10miR mosquitoes showed lower, but not significant, infection and dissemination rates (Fig. 5). The CHIKV transmission-reducing phenotypes of the transgenic mosquitoes were also confirmed by a salivary glands using an immunofluorescence assay. Salivary glands dissected at 6 dpi, reacted with antibodies and visualized under fluorescent microscope, showed qualitatively lower signals in samples from each transgenic line than wild-type Orlando mosquitoes (Supplementary Figure 5). Anti-DENV-3 phenotypes tested at 21 dpi showed that the infection rate, dissemination and transmission efficiencies of the transgenic lines were significantly lower than wild-type mosquitoes (Fisher’s exact test: *p* < 10^−4^) (Fig. 5).

## Discussion

Against arboviruses, exogenous RNAi induced by long dsRNA molecules is an effective mechanism to interrupt viral infection and transmission^46^. Several studies have demonstrated the highly effective antiviral siRNAs in genetically engineered mosquitoes. For example, long dsRNAs (>500 bp in length) derived from DENV-2, were processed into several siRNAs targeting the viral genome and suppressed viral replication^35,47^. The high coverage of the antiviral siRNAs on the viral genome provides a high level of resistance against DENV-2 and reduces the risk of generating siRNA escape variants. However, owing to the large diversity of antiviral siRNAs produced, it is difficult to predict regions targeted in the mosquito transcriptome^48^. The effects of RNAi machinery employing siRNA to suppress viral replication can be transient as some viruses replicate so quickly that they overcome the RNAi response^49^. These limitations can be surmounted using hammerhead ribozymes. In cells and genetically engineered mosquito experiments, the small catalytic hammerhead ribozymes mediate a 15–16 nt sequence-specific cleavage and are efficiently used as an antiviral effector against CHIKV, which increases the range of possible target sites^50^. However, the error-prone activities of RNA polymerase generate opportunities for arboviruses to escape from ribozyme catalysis, which is only triggered under high sequence specificity^51,52^. This deficiency can be overcome by using antiviral group-I introns^53,54^ and by targeting the conserved DENV and CHIKV sequences, which then could lead to viral RNA trans-splicing and cell apoptosis. So the resistance to arboviruses could be triggered by incomplete viral RNA synthesis and cell death. Targeting the conserved viral sequences can successfully increase the coverage of the four serotypes of DENV and CHIKV, without inducing significant fitness impacts in naïve C6/36 cells. The antiviral activities of group-I introns were initiated after recognition of several components mediating RNA splicing, including internal guide sequence, external guide sequence, and a helix forming sequences (P10) on both viruses. However, the unknown mismatch tolerance of antiviral group-I introns might favor escape variants due to the quasi-species nature of viral populations. Cell-based experiments may not reflect the complexity of a mosquito organism, so the fitness impact needs to be examined carefully in mosquitoes. In addition, the antiviral apoptotic cell death that is triggered upon viral infection might result in different outcomes depending on the virus. Apoptosis can suppress DENV replication but not SINV in mosquitoes^55,56^. Nevertheless, if a few mismatches between the guide sequence and the target virus can be tolerated, the antiviral group-I introns system in mosquitoes is a potentially applicable molecular effector against arboviruses.

In this study, we generated several miRNA-based genetically engineered mosquito lines with resistance to DENV-3 and CHIKV triggered either ubiquitously or midgut specifically in responding to a blood meal. The synthetic miRNAs we used were 22-nt in length, which are capable of targeting a broad range of viral strains. Additionally, the predictable off-target effect of antiviral miRNA provides tolerable features with mutant variants that reduce the risk for the virus to escape from miRNA-mediated silencing. In addition, the small sized synthetic miRNAs with distinct targets can be easily assembled and transcribed as a miRNA cluster, then processed into mature miRNAs through endogenous miRNA pathway, without eliciting unintended silencing resulting from siRNAs derived from long dsRNA.

Although the recipient mosquito strain Orlando was reported as weakly susceptible to DENV-2^57^, its vector competence depends on the virus^58^. Orlando mosquitoes are still susceptible to DENV-3 when provided at a viral titer of 1 × 10^7^ ffu/mL.

Insertions of transgenes and their subsequent expression may impose a load on the mosquitoes carrying them. This load could result in a fitness cost for the transgenic lines and may impair their ability to be used in control strategies. There are several previous reports of exogenous gene expression causing a variety of effects on transgenic mosquitoes^59,60^. We observed a significant effect in AeCPA > 10miR compared to wild-type mosquitoes at immature and adult stages: longer larval development time, higher larval and pupal mortalities, lower adult survival, lower proportion of females at emergence and lower male mating competitiveness. These effects may be caused by the strongly expressed reporter DsRed in the AeCPA > 10miR mosquito line. Nevertheless, AePUb > 10miR mosquitoes did not share the same effects. In contrast, they had a shorter larval development time, lower larval and pupal mortalities, higher adult survival, higher proportion of females at emergence and higher male mating competitiveness, all of which could facilitate vector control^59^. Although a distinct result was observed between both mosquito lines, we were not able to conclude that the AePUb > 10miR construct has a lower fitness impact to mosquitoes than the AeCPA > 10miR construct, as they do not share the same insertion site and this may cause some bias. The fitness tests in this study could only provide additional information for the two selected lines carrying their distinct antiviral construct.

For testing the viral reduction phenotype under AePUb and AeCPA induction strategies, we examined co-infected mosquitoes with CHIKV at 6 dpi and DENV-3 at 21 dpi during the plateau phase of viral replication in *Ae. aegypti*^61^. Saliva titers of DENV-3 and CHIKV were reduced in both transgenic mosquito lines; however, viral infection and dissemination were not impaired compared to wild-type mosquitoes. For CHIKV, although transmission efficiencies were reduced in both transgenic lines, AePUb > 10miR and AeCPA > 10miR mosquitoes showed a lower but not significant difference in infection rate and dissemination efficiency suggesting that only salivary glands but not midguts behave as an efficient barrier to the release of the virus in saliva. In contrast, the transgenic mosquitoes showed more promising results on DENV-3 suppression than CHIKV when examining infection, even though only four regions on DENV-3 were targeted by our anti-DENV-3 miRNAs.

It is likely that the anti-DENV-3 miRNAs have higher silencing efficiency than anti-CHIKV miRNAs, or the expression levels of anti-CHIKV miRNAs were not sufficient to suppress CHIKV characterized by a shorter extrinsic incubation period^61–63^. To overcome this issue, replacing the miRNA targeting regions or substituting the promoter with other high-activity promoters would be a solution for optimizing the miRNA-based mosquitoes^64,65^. In addition, the different expression patterns of antiviral miRNAs might cause different viral reduction phenotypes for each mosquito line. Although the characteristics of *Aedes PolyUbiquitin* and *Carboxypeptidase A* gene promoters are well studied^66,67^, the position of transgene integration also could be important in determining antiviral potential^68^.

In summary, we successfully demonstrated the feasibility of using artificial antiviral miRNAs to reduce the transmission of two major arboviruses in transgenic *Ae. aegypti*. Although most of the genetically engineered mosquito lines are still able to transmit DENV-3 and CHIKV, the DENV-3 transmission rates were reduced by 94.16% in AePUb > 10miR mosquitoes (from 23.61% to 1.38%), and the CHIKV transmission rates were reduced by 77.33% (from 41.67% to 11.11%) and 83.35% (from 41.67% to 6.94%) in AePUb > 10miR and AeCPA > 10miR mosquitoes, respectively. These reductions would greatly limit the virus circulation. However, the effector has to be optimized to approach 100% of viral suppression at midgut infection level, eliminating the risk of virus dissemination. To apply these genes in a population replacement strategy, they should be combined with a gene-drive system, such as Cas9-mediated or toxin-antidote underdominance gene drive system, by introgressing the homozygous antiviral effector gene into target wild populations to reduce disease transmission^69,70^. Therefore, maintaining high-viral suppression efficiency with low fitness impacts after combining with mosquito gene drive system is needed. Thus, the mosquitoes that we presented in this study are not yet applicable in the field and viral suppression at infection level should be improved. For mosquitoes that are released in the field, a “localized” transgenic line for control program is also needed. A local mosquito strain must be used for mosquito transgenesis, to reduce the alteration of the population’s gene pool. Therefore, the fitness issue of released mosquitoes should be analyzed again for determining the replacement efficiency in the target population. In this study, we have shown the potential of using synthetic antiviral miRNAs as effector genes to combat multiple arboviruses simultaneously. As the proof-of-concept has been validated, we can extend our strategy to other *Aedes* mosquito-borne arboviruses such as YFV and ZIKV.

## Methods

### Plasmid DNA constructions

All the plasmids in this study were generated based on the backbone of pMOS1_nanos-mimyd88_3xp3-CFP originated from Dr. Bruce A. Hay (Caltech, CA), and re-modified by replacing the *Bgl*II site upstream of tub 3′ UTR with *Bam*HI/*Xho*I sites (underlined) using PCR primers tub-3′UTR_ BamHI/XhoI-F and SV40-3′UTR_ NotI-R (Supplementary Table 4), generating pMOS1_nanos-mimyd88_3xp3-CFP′. The anti-DENV-3 and anti-CHIKV miRNA stem-loop backbones containing 5′-EcoRI/BglII and 3′-XhoI/BamHI were generated by oligo synthesis, and subcloned into pMOS1_nanos-mimyd88_3xp3-CFP with *EcoR*I and *Xho*I sites, generating pMOS1_nanos-Den3-4miR_3xp3-CFP and pMOS1_nanos-CHIKV-6miR_3xp3-CFP. The AePUb promoter was amplified from *Ae. aegypti* genomic DNA by PCR primers pMOS1_fusion_FseI/PstIAePUb-pr-F and pMOS1_fusion_BglII/EcoRIAePUb-pr-R, and subcloned into *Fse*I and *EcoR*I double digested pMOS1_nanos-Den3-4miR_3xp3-CFP and pMOS1_nanos-CHIKV-6miR_3xp3-CFP with In-Fusion^®^ HD Cloning technology (Clontech), generating pMOS1_AePUb-Den3-4miR_3xp3-eGFP (GenBank accession: MG603748)and pMOS1_AePUb-CHIKV-6miR_3xp3-eGFP (GenBank accession: MG603749).

DENV-4miR was extracted from *EcoR*I and *BamH*I double digested pMOS1_AePUb-Den3-4miR_3xp3-CFP, and CHIKV-6miR was extracted from *Bgl*II and *Xho*I double digested pMOS1_AePUb-CHIKV-6miR_3xp3-CFP. The two antiviral miRNA clusters were then subcloned into *Bgl*II and *Xho*II double digested pMOS1_AePUb-Den3-4miR_3xp3-CFP, generating pMOS1_AePUb-Den3-CHIKV-10miR_3xp3-eGFP (GenBank accession: MG603750). AeCPA promoter was amplified from *Ae. aegypti* genomic DNA by PCR primers pMOS1_fusion_FseI/PstIAeCPA-pr-F and pMOS1_fusion_BglII/EcoRIAeCPA-pr-R, and then subcloned into *Fse*I and *EcoR*I double digested pMOS1_AePUb-Den3-CHIKV-10miR_3xp3-CFP, generating pMOS1_AeCPA-Den3-CHIKV-10miR_3xp3-GFP. This plasmid was then re-modified by replacing the reporter 3xp3-CFP with *Bgl*II and *Xho*I sites (bolded) disrupted Hr5IE1-DsRed which carried out by In-Fusion^®^ HD Cloning technology with mutation primers Oxitec_#4573_BglII-mutate-F and Oxitec_#4573_XhoI-mutate-R. By using In-Fusion^®^ HD Cloning technology with PCR primers pMOS1_fusion_Hr5IE1-DsRed_marker_NotI-F and pMOS1_fusion_Hr5IE1-DsRed_marker_XmaI-R, the Hr5IE1_DsRed was subcloned into *Not*I and *Xma*I double digested pMOS1_AeCPA-Den3-CHIKV-10miR_3xp3-CFP, generating pMOS1_AeCPA-Den3-CHIKV-10miR_Hr5IE1-DsRed (GenBank accession: MG603751).

### miRNA off-target effect examination

The off-target effect of synthetic antiviral miRNAs was predicted by using GESS (version 1.2) with the input parameters as followed, 7 nt of siRNA seed sequence to test; Minimum 1 of seed matches to consider an siRNA Seed Matching; Guide and Passenger strands were used for analysis; P1C-seeds of active siRNAs as inactive siRNA seeds were used; All siRNA seed sequences were scrambled as GESS control; No siRNA exclusion was allowed; *p*-value 0.05 was set as significance threshold parameter; Benjamini & Hochberg False Discovery Rate was selected for testing correction.

### Generation of transgenic mosquitoes

*Ae. aegypti* Orlando strain was used as the recipient for germ-line transformation; the preblastoderm embryos were injected with the mixture of donor and helper plasmids at a ratio of 300:500 ng/µL in injection buffer (5 mM KCl and 0.1 mM NaH_2_PO_4_, pH 6.8). Mosquitoes were reared at 28 °C, 70% relative humidity, and a 12:12 light/dark regime and fed ad libitum with a 10% sucrose solution.

Embryo microinjection was carried out as described in Lobo et al.^44^. Each surviving G_0_ male adult was outcrossed with 3 wild-type females, G_0_ females were pooled together and crossed with wild-type males at a male/female ratio 1:3. All the eggs collection and G_1_ larvae screening were carried out individually. G_1_ larvae were screening for reporter gene expression under a fluorescent microscope (LeicaMZ12.5, Wetzlar, Germany). The transgenic G_1_ mosquitoes were then outcrossed with wild-type mosquitoes for one generation to confirm the Mendelian inheritance in progenies. To establish homozygous lines, the transgenic mosquitoes were inter-crossed individually and the homozygous candidates were screened for two generations.

### Mosquito experimental infections

Seven-day-old female adults were fed on artificial infectious blood meal containing 1.4 mL of washed rabbit red blood cells and 0.7 mL of virus infected C6/36 cells suspension. The blood meal was supplemented with ATP as a phagostimulant at a final concentration of 1 mM and provided to mosquitoes using a Hemotek membrane feeding system. Virus titers of the artificial infectious blood meal were at 10^6^ and 10^7^ ffu/mL for CHIKV and DENV respectively. Engorged mosquitoes were transferred into cardboard containers and maintained with 10% sucrose under a photoperiod of 12:12, at 28 °C. Mosquito saliva was collected using the forced salivation technique described in Dubrulle et al. (2009)^61^. After removing mosquito wings and legs, the proboscis was inserted into P20 tips filled with 5 µL of fetal bovine serum (FBS). After 30 min, saliva was expelled from the tip to 45 µL of L-15 medium. After salivation, mosquito head and body were collected and grounded individually in 300 µL of L-15 medium supplemented with 2% FBS. In total 200 µL of homogenates were collected for titration after centrifugation at 10,000 × *g* for 5 min.

Mosquitoes were examined at 6 dpi when infected with CHIKV and 21 dpi with DENV-3. Infection rate (IR) refers to the proportion of mosquitoes with infected body among engorged mosquitoes. Dissemination efficiency (DE) corresponds to the proportion of mosquitoes with infected head among mosquitoes with infected body. Transmission efficiency (TE) represents the proportion of mosquitoes with infectious saliva among mosquitoes examined.

### Mosquitoes screening test

To avoid any bias caused by the position effect of integration on antiviral efficiency, a mosquito screening test was conducted with three independent lines of AePUb > 10miR and five independent lines of AeCPA > 10miR mosquitoes (Supplementary Table 2), and the virus transmission efficiency was analyzed to select the mosquito lines that exhibited the strongest antiviral phenotype for each construct (Supplementary Figure 6). Mosquitoes were co-challenged with both DENV-3 and CHIKV as described above, and the saliva were collected at 6 and 14 dpi for analyzing virus transmission efficiency. The two selected lines for each antiviral construct were used for further analysis.

### Southern blot analysis

In total 20 µg of genomic DNA was produced and digested with restriction enzymes *Bgl*II or *Sca*I, followed by DNA separation on 0.8% agarose gel. The separated DNA was then transferred onto a nylon membrane and hybridized with random-primed [α^32^P] dCTP-labeled DNA probes complementary to the 10miRNA clusters at 42 °C for 16 h. No restriction enzyme site of *Sca*I was present in the transgene and only one *Bgl*II site was present in the upstream of miRNA cluster, which makes the expected size of hybridization patterns»6698 bp and >>7591 bp for *Sca*I; >>4927 bp and >>6079 bp for *Bgl*II digested AePUb > 10miR and AeCPA > 10miR mosquitoes, respectively.

### Virus detection in salivary glands

The viral particles of CHIKV were detected in mosquito salivary glands by immunofluorescence assay. Salivary glands were dissected in PBS and fixed with 4% paraformadehyde at 6 and 21 dpi, followed by hybridization with anti-CHIKV antibodies respectively. After exposing to secondary antibodies, tissues were transferred on a slide with mounting solution (ProLong^®^ Gold Antifade Mountant). The infection patterns were visualized under fluorescent microscope. DAPI was used for cell localization.

### Life-table parameters of transgenic mosquitoes

Seven-day old eggs were vacuum hatched to synchronize the rearing process. Newly hatched larvae were counted and reared in daily renewed 1 L of water with 1 yeast tablet. Larvae were checked daily until pupation and adult emergence. In total 50 adults of each sex from the same batch of mosquitoes were pooled together and maintained on 10% sucrose for adult lifespan analysis. For mating competitiveness test, 20 virgin females of wild-type mosquitoes were grouped with 10 wild-type males and 10 transgenic males. Mosquitoes were fed on blood meal at 7 days after being grouped up, and eggs were collected from each female on 4 days after blood meal. All mosquitoes were reared at 28 °C and 70% in relative humidity with a photoperiod of 12:12.

### Artificial miRNA expression analysis

Mosquito small RNA was extracted from the midguts and carcasses of sugar fed and 24 hPBM mosquitoes. Tissues were lysed in Trizol solution and total RNA was precipitated with 75% ethanol (v/v). Total RNA was used for miRNA cDNA synthesis by MystiCq^TM^ microRNA cDNA Synthesis Mix (Sigma-Aldrich), whereas qPCRs were performed using MystiCq^®^microRNA^®^SYBR^®^ Green qPCR Ready Mix^TM^ (Sigma-Aldrich) on Applied Biosystems 7500 Fast. Primer sequences are included in Supplementary Table 3. aae-miR-1 is one of the most highly and relatively stably expressed miRNA in *Ae. aegypti* and was used as an internal control for detecting miRNAs expression in this study. Because artificial miRNAs were not expressed in wild-type mosquitoes, data were normalized twice to each aae-miR-1 and wild-type aae-miR-1 presenting the relative expression profile.

### Statistical analysis

All statistical tests were conducted using the STATA software (StataCorp LP, Texas, USA). Proportions were compared using Fisher’s exact test and sample distributions with the Kruskal–Wallis test. *P*-values>0.05 were considered non-significant.

### Data availability

All data generated or analyzed during this study are included in this published article (and its supplementary information files). The accession codes of the plasmid sequences are MG603748 (pMOS1_AePUb-Den3-4miR_3xp3-eGFP), MG603749 (pMOS1_AePUb-CHIKV-6miR_3xp3-eGFP), MG603750 (pMOS1_AePUb-Den3-CHIKV-10miR_3xp3-eGFP) and MG603751 (pMOS1_AeCPA-Den3-CHIKV-10miR_Hr5IE1-DsRe).

## Electronic supplementary material

Supplementary Information

Supplementary Data 1

Description of Additional Supplementary Files
