# Supplementary material for: Synthetic miRNAs induce dual arboviral-resistance phenotypes in the vector mosquito Aedes aegypti
Source: Commun Biol. 2018 Feb 8;1:11. doi: 10.1038/s42003-017-0011-5 (PMC6053081; doi:10.1038/s42003-017-0011-5)

Supplementary Table 1. Details on artificial antiviral miRNAs

miRNA name	Sequence (5' – 3')	Targeting virus	Targeting region	Coverage (%)
Anti-CHIKV-1	AGTCAGTTCTGCTTCTCGTTCT	CHIKV	NSP1	96.9
Anti-CHIKV-2	ACTCATTCGTAGTGCGCATTTT		NSP2	96.9
Anti-CHIKV-3	TATATACCCACCTGCCCTGTCT		NSP3-NSP4	96.9
Anti-CHIKV-4	TCTATGATCTTCACTTCCATGT		NSP4	100
Anti-CHIKV-5	ACTCTTCTTGATAGTTTGGTTC		E2	96.9
Anti-CHIKV-6	GTTTTGCATGATTCGGACTTCT		E2	96.9
Anti-DENV3-1	TCTCATTGTTCCATCATCATCA	DENV-3	NS2B	96.6
Anti-DENV3-2	CCTGTGTGTTCAGATTTTGTTG		NS3	96.9
Anti-DENV3-3	AATATGACCAGCCTCCTCTTCC		NS3	98.6
Anti-DENV3-4	CATTTATCATGGAGGAGGCTGA		NS5	97.2

Supplementary Table 2. Performances of *Aedes aegypti* microinjections

Construct	Eggs injected	Survival to larval stage	G0 adults		Integration events
			Male	Female	
PUb>4miR:DENV-3	432	187	81	70	1
PUb>6miR:CHIKV	595	153	85	68	5
PUb>10miR	310	153	76	60	3
CPA>10miR	185	85	11	12	5

Supplementary Table 3. Primers used for miRNA qPCR in this study

aae-miR-1:	5'-TGGAATGTAAAGAAGTATGGAG-3'
10miR_DENV-3:	5'-TCTCATTGTTCCATCATCATCA-3'
10miR_CHIKV:	5'-TCTATGATCTTCACTTCCATGT-3'

Supplementary Table 4. Details on primers used for constructing synthetic antiviral miRNA cassettes.

Oligo name	Sequence (5' - 3')
tub-3'UTR_ BamHI/XhoI-F	GGCGGATCCCGCCCTCGAGATCCGTCACAAGCAATCTCACTA
SV40-3'UTR_ NotI-R	GGCCGCGGCCGCGACTCTAGATCATAATCAGCCATAC
pMOS1_fusion_FseI/PstIAePUb-pr-F	GACGAGATCGGCCGGCCCGCCCTGCAGTATCTTTACATGTAGCTTGTGCATTGAATCC
pMOS1_fusion_BglII/EcoRIAePUb-pr-R	AGATCTGGCCGAATTCGTTGAAATCTCTGTTGAGCAGAAAAAGAAACGAG
pMOS1_fusion_FseI/PstIAeCPA-pr-F	GACGAGATCGGCCGGCCCGCCCTGCAGATACATAAACTAGTTTTTGCACA
pMOS1_fusion_BglII/EcoRIAeCPA-pr-R	AGATCTGGCCGAATTCTCCAACTAACCGATACACACTAACCTGG
Oxitec_#4573_BglII-mutate-F	TCTTGGGTCGAGAGC**G**CAGGAACAGGTGGTGGCGGCCCTCGGTGCGC
Oxitec_#4573_XhoI-mutate-R	CAAACGGACGC**C**CCGAGGTTGCACAACACTATTATCGATTTGC
pMOS1_fusion_Hr5IE1-DsRed_marker_NotI-F	TCTAGAGTCGCGGCCGCTCCGGTGGATCTTACGGGTCCTCCACCTTCCGC
pMOS1_fusion_Hr5IE1-DsRed_marker_XmaI-R	TTCGAGCTCGCCCGGGCATTGCTTGTCATTTATTAATTTGGATGATGTCA

Supplementary Figure 1. Southern blot analyses of transgenic mosquitoes. Genomic DNAs were digested with restriction enzyme *Bgl*II or *Sca*I, and hybridized at 42°C with random primed alpha [^{32}P]-labeled DNA probes complementary to the sequence of the antiviral miRNA cluster.

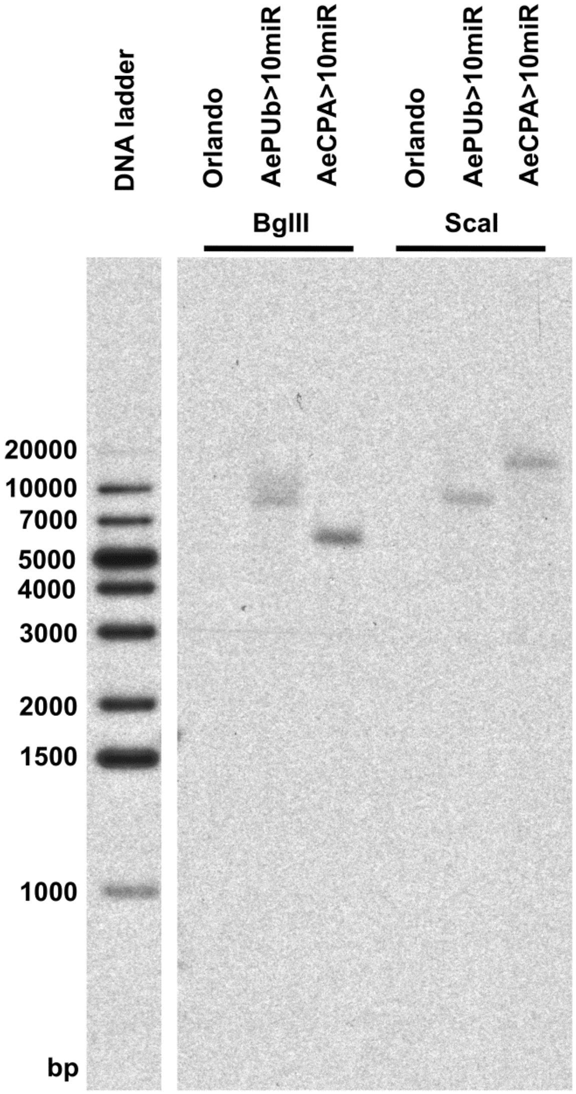

Supplementary Figure 2. Detection of artificial antiviral miRNAs. a in midgut and carcass. **b** in salivary glands. Information on replicates is provided and details are described in the legend of the figure 2.

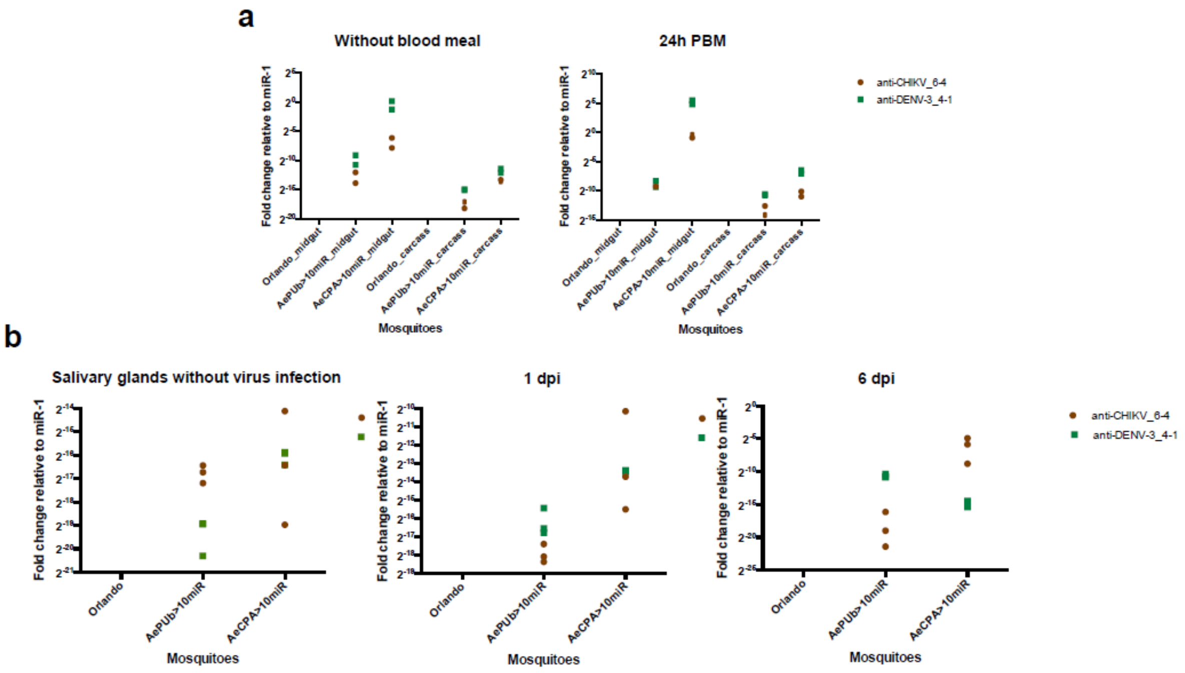

Supplementary Figure 3. Anti-DENV-3/CHIKV phenotype of transgenic 4miR:D3 and 6miR:Chik mosquitoes. a All samples. **b** Day 6 post CHIKV-infection. **c** Day 14 post DENV-3 infection. **d** Day 21 post DENV-3 infection. Information on replicates is provided and details are described in the legend of the figure 4. R1, replicate 1. R2, replicate 2

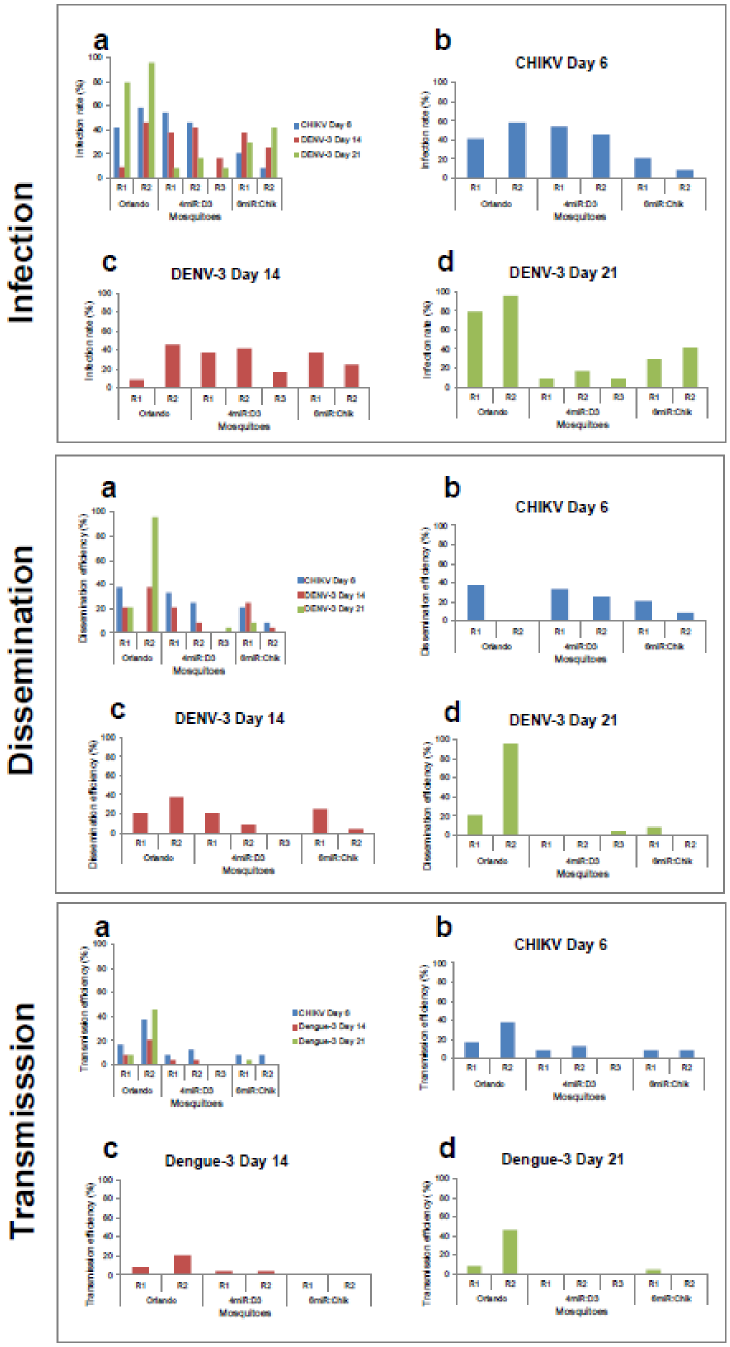

Supplementary Figure 4. Anti-DENV-3/CHIKV phenotype of transgenic AePUb>10miR and AeCPA>10miR mosquitoes. a All samples. **b** CHIKV. **c** DENV-3. Information on replicates is provided and details are described in the legend of the figure 5. R1, replicate 1. R2, replicate 2. R3, replicate 3.

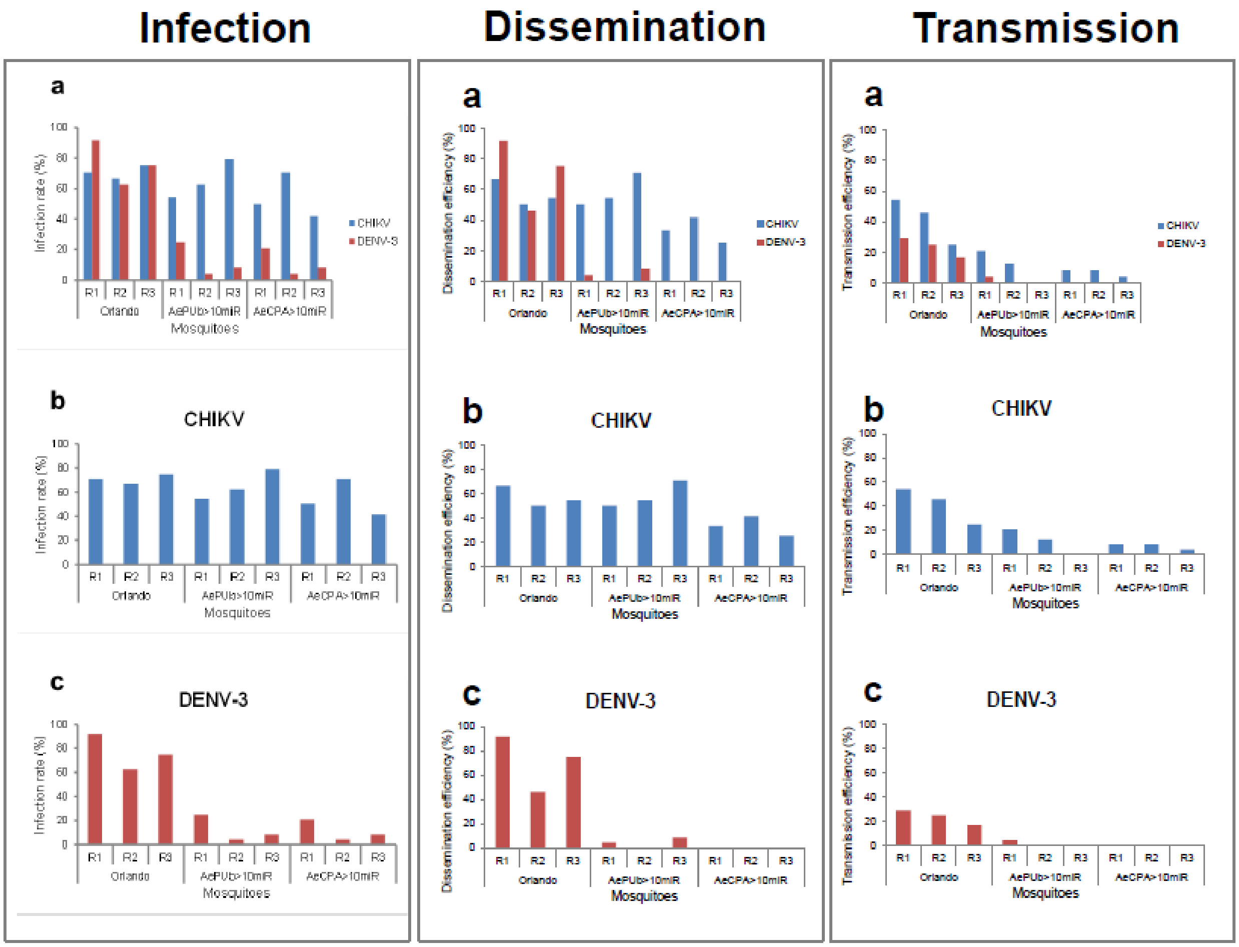

Supplementary Figure 5. Antiviral phenotype in salivary glands. Mosquito salivary glands were dissected in PBS and fixed with 4% paraformadehyde at 6 days post-infection, followed by detection with anti-CHIKV antibody. The viral infection patterns were visualized under fluorescent microscopy.

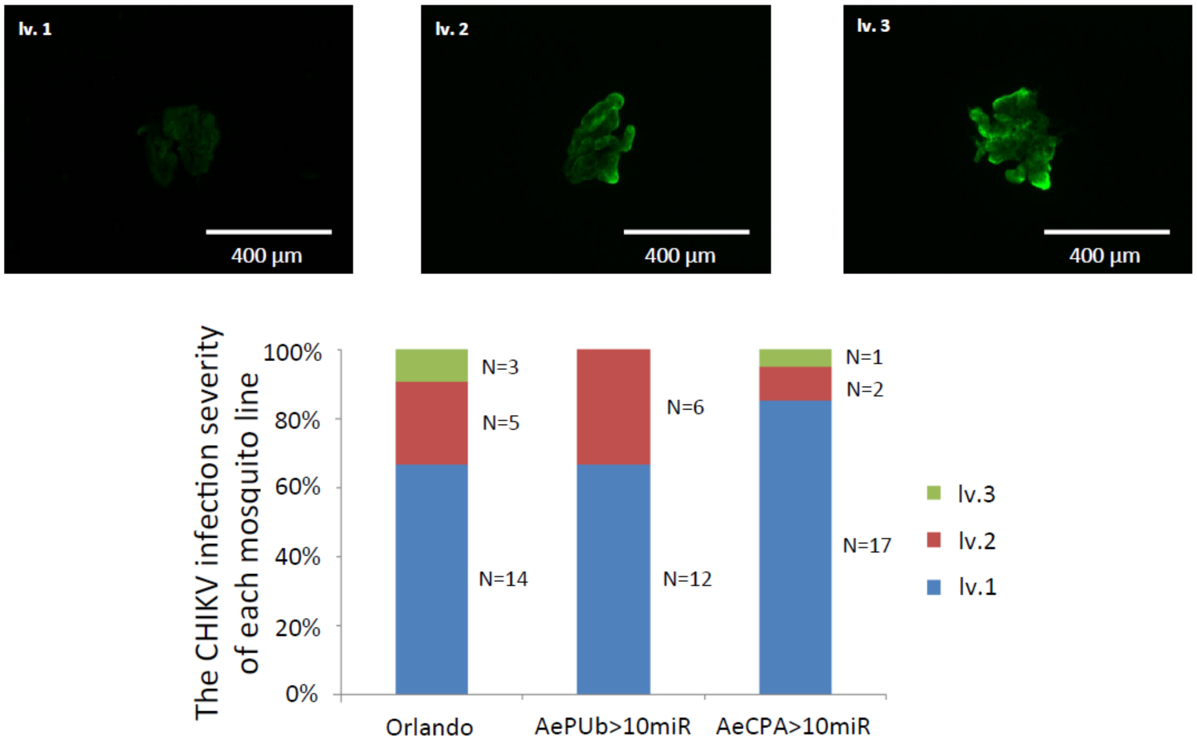

Supplementary Figure 6. Antiviral phenotypic screenings for AePUb>10miR and AeCPA>10miR mosquito lines. The mosquito lines AePUb>10miR and AeCPA>10miR were co-challenged with DENV-3 at 10^7 ffu/ml and CHIKV at 10^6 ffu/ml. The viral suppression efficiency was determined by the transmission efficiency at 6 and 14 dpi for CHIKV and DENV-3 respectively. AeCPA>10miR-3 (AeCPA>10miR) and AePUb>10miR-1 (AePUb>10miR) were analyzed in this study. Numbers above are the transmission efficiency and sample size.

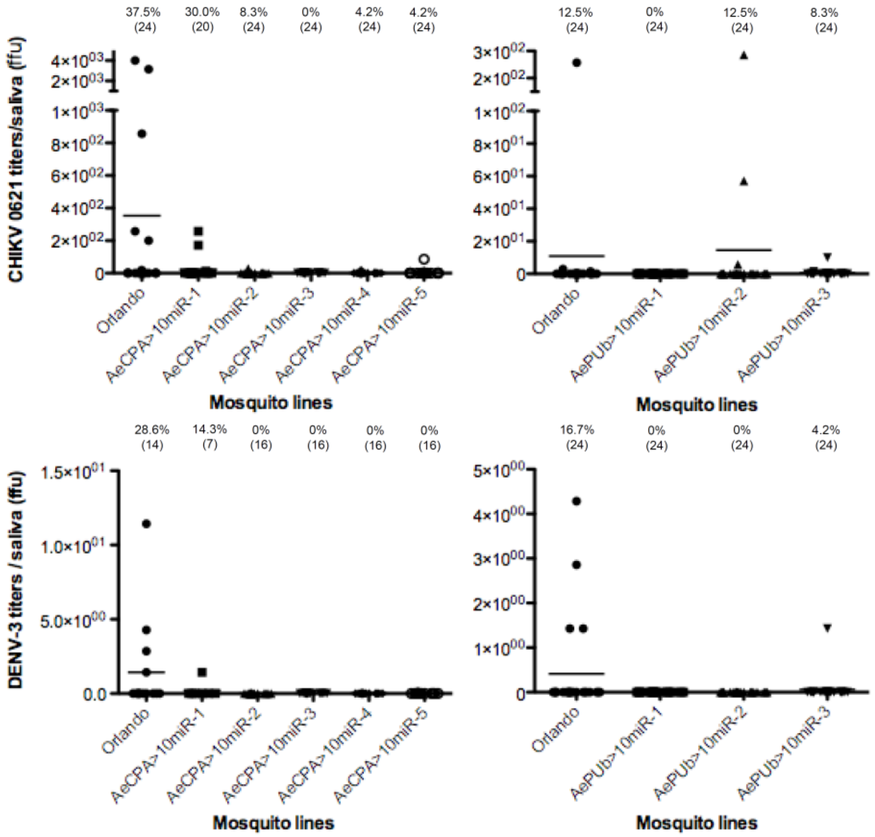

Supplement: Supplementary file 1 — Supplementary Information [file 42003_2017_11_MOESM1_ESM.pdf]
