# Supplementary material for: Synthetic miRNAs induce dual arboviral-resistance phenotypes in the vector mosquito Aedes aegypti
Source: Commun Biol. 2018 Feb 8;1:11. doi: 10.1038/s42003-017-0011-5 (PMC6053081; doi:10.1038/s42003-017-0011-5)
Supplement: Supplementary file 2 — Supplementary Data 1 [file 42003_2017_11_MOESM2_ESM.docx]

**Supplementary Data 1. Consensus sequences.** **a** DENV-3. **b** CHIKV. Consensus sequences based on 356 strains of DENV-3 and 32 strains of CHIKV. Bold and underlined regions are the binding sites of antiviral miRNAs.

**a**

akttgttagttctvcgtggaccgacaagaacagtbttgcwgtttcaayttcggaaagctctgctttaacgtagtrctracagttttttattagagagcrgatytctgatgaacaaccaacgraaraagacgggaaaaccgtctatcaatatgctgaaacgcgtgagaaaccgtgtgtcaactggatcacagttggcgaagagattctcaaraggaytgctgaayggccarggaccaatgaaaytggthatggcgttcatagctttcctcagatttctagccattccaccracagcrggartbttggctagatggggaaccttyaagaagtcrggggcbatyaaggtcctdaraggcttcaagaaggagatytcaaayatgctgagcathatcaacaramgraaaaagacatcghtctgyctcatgatgatrhtvccagcarcacttgctttccacytgacttcacgngatggagagcctgcgcatgattgtggggaagaatgaaagaggdaaatcchtacttttyaagacagcytctggaatyaacatgtgcacactcatagcyatggaytytrggagaratgtgtgatgacacggtcacttayaaatgcccccwyattrcygaagtggarcctgaagacattgaytgctggtgcaacctyacatcracrtgggtgachtayggaacgtgcaaycaagcyggrgagcayagacgcgayaagagatcrgtrgcgttrgctccccatgtyggcatgggactrgacacacgyacccaaacctggatgtcrgctgaaggagcttggagrcaagtygaraaggtagaracatgggcyyttaggcacccaggkttcaccaatactagcyctattycthgcccattacataggcachtccytgacccagaargtggthattttyrtactaytaatgctggtcacyccatccatgacaatgagatgygtgggagtaggaaacagagattttgtggaaggnctrtcrggagchacgtgggttgaygtggtgcthgarcacggdgggtgtgtgacyacyatggctaagaacaagccnacgytggayatagagctycagaagaccgaggccacccaaytggcdaccctaaggaarytatgcattgagggdaaaatyaccaacrtracaacygactcaagrtgyccyacccarggggaagcrrytytrcctgaggagcaggaccagaactacgtrtgtaagcayacmtacgtdgayagaggytggggraacggdtgtggyttgtttgghaarggaagcttggtdacatgygcdaaatttcaatgyytrgaaycaatagagggaaaagtggtgcaryatgagaacctyaaatacacbgtyatcatyacagtgcacacaggagaytcaacaccargtgggaaatgahacgcarggagtcacggytgagataacacchcaggcatcaacccryygaagcayrtcttrcctgartatggaacccttgggctagaatgytcaccacggacaggyttggayttyaatgaaatgatyytrytracaatgaaraacaaagchtggatggtacatagrcaatggttytttgacctaccyctaccatggrcatcaggrgchacaacrgaracaccaacytggaayargaargagcttcttgtracattyaaaaaygcacaygcraaraarcaagaagtrgtngtccttggatcgcaagagggagcaatgcayachgcdytgacaggagcyacagaratycaaamctcaggagghacaagyatyttygcggggcayttraaatgyagactyaagatggacaaattrgaactcaaggggatgagctatgcaatgtgcwyraatrccttygtgttgaagaargaagtctcngaaacrcarcaygggacaatactcathaaggtygagtacaaaggggaagatgyaccttgcaagattcchttytccacrgaggatggacaagggaaagcycacaatggyagaytgatyacagchaacccwgtggtgacyaagaaggaggagcctgtcaayattgaggcdgaacctccttttggggaaagyaayatartrattggaattggagacaaacgccttgaaaatyaaytggtayaagaarggragctcnattgggaagatgttygaggccactgchagrggygcaaggcgcatggccatcttgggagacacagcntgggactttggwtcagtrggtggtgttytraaytcaytaggnaaaatggtgcaccaaatattyggaagtgcytacacrgcacctrttytagyggagtctchtggrtratgaaaathggaataggdgtyctyttracbtggatagggttgaattcaaaraacachtcyatgtcattttcatgcattgygataggaatyatyacactctatctgggagcygtggtrcaagctgacaygggdtgygybataaactggaargghaaagaactcaaatgtggragyggaatyttygtcacyaaygaggtccayacctggacagagcaatacaaattycaagcagaytccccnaaaagabtggcvacagccatygcaggcgcbtgggaraatggagtntgyggaatyaggtcracaaccagaatggagaayctmytgtggaagcaaatagccaatgarctraactacatattrtgggaaaacaayatcaaattaacggtagttgtgggygayayaattggggtcttagagcaaggraaaagaacaytaacaccacarcccatggagctaaaataytcatggaaaacrtggggaaaggcraaaatagtgacagctgaaahacaaaattcytcyttyataatagayggdccaaacacaccrgagtgtccaartgcctcaagagcatggaatgtgtgggaggtggaagattacgggttyggagtyttcacaacyaayatatggctgaaactccgagagrtgtayacccaawctatgygaccayaggctaatgtcggcagcyrtyaargatgagagrgcygtrcaygccgacatgggctaytggatagaaagccaaaagaatggdagttggaagctagaaaargcatcyytcatagaggtgaaaacctgyachtggccaaaatcacayacyctytggagyaatggtgtvytrgagagtgacatgatcatyccaaagagtctdgctgghccyatytcrcaacacaacyacaggccyggrtaccacacccaracggcrggaacchtggcayytrggaaaattrgagctggacttyaactaytgygaaggaacaacagthgtcatcacagacaarytgtgggacaagagggcccatcaytgagracaacaacagtgtcagggaagytratacacgaatggtgytgccgytcgtgyacacttccycccytrcgatacatgggagadgacgghtgctggtatggcatggaaatyagacchatyartgagaaagaagagaayatggtaaagtctttagtctcagcrggragtggaraggtggayaacttcacaatgggwgtcytgtgtcttggcaatcctcttygaagaggtratgagaaggaaaattygggaaraarcacatgattgcrggggtathtcttyacdtttgtrctccttctctcagggcaaataacatggagagayatggcrcrcacactmataatgatyggrtccaacgchtctgacagratgggaatgggcgtyacytacytrgcatytrattgcaacattyaaaathcagccattyttggctttgggattyttcctvagraarytgacatcyagagaaaatttrytrttrggagttgggytggcyatggcaacaacgttrcaactgccagaggacattgaacaaatggcraatggaathgcyytggggctcatgrctctnaaaytgataacacaatttgaracataycarytrtggacrgcattrrtctccytaayrtgttcaaayacaatbttyacgytgactgttgcctggagaacagccacyytgattytggccggartttcgcttttdccabtgtgccagtcttcragcatgaggaaawcagaytggctnccaatgrcngtggcagchatgggagttccaccgyytwccactttttatyttyagyttraargayacacyyhaaaaggagragctggccactgaatgarggrgtgatggchgttggvcttgtgagcattctrgchagttctctccttagraatgaygtrcccatggctggaccaytagtggcygggggcttgytgatagcgtgctaygtyataactggcacgtcagcdgacctcacygtagaaaargcagcagatrtracatgggaggaagargctgagcaracaggagtgtcccacaayttratgatcacagt**tgatgatgatggaacaatgaga**ataaargatgatgagactgagaayatyyctaacagtgctyttraaaacagcattactaatagtdtcaggcartytttccatactccathccygcnacahtgytggtctggcayacttggcaaaarcagaacccraagatcyggcgtyytatgggaygtnccyagcccyccagaracacagaaagcagaactggaagadggggthtataggatcaavcarcaaggaatttttggraaracccaagtdggggttggagtrcadaaagaaggagtyttycacacyatgtggcacgtyacaagaggrgcagtrttracryayaatgggaaaagactggaaccaaactgggchagygtgaaaaaagatctgatttcatayggaggaggatggagaytgagygcacatatggcaraarggrgargaggtgcaggttattgccgtagagcchgggaagaacccaaaraacttycaaaccatgccrggcaytttycagacnacnacrggggarataggagcrattgcactrgayttcaagcctggrachtcaggatctccyatcataaayagagagggaaaggtagtgggactrtatggcaatggagtggtyacdaaraatggtggctaygtcagyggaatagcdcaaacraaygcagaaccagayggaccgacaccrgarttrgaagaagaratgttcaaaaarcgaaahctracyataatggatctncatccyggrtcaggaaagacrcggaaataycttccagcyathgtyagagargcaatcaagagacgyttragracyytaattytggcaccracaagrgtrgttgcagctgagatggaagaagcahtgaaacggrctcccaataaggtaycaaacaachg**caacaaaatctgaacacacagg**rarrgagattgttgatctratgtgycacgcaacrttcacaatgcgyytgctrtcaccagtyagrgtyccaaaytayaayytgataataatggatgargcycatttcacagayccagccagyatagckgctagagggtacatatcractcgtgtnggaatgggagargcagchgcaathttcatgacagchacrccccctggaacwgctgahgcctttccycagagcaaygctccaatycaagatgaagaaagrgayathccrgaacgctcatggaattcaggcaatgahtggathaccgacttygyhggraaracdgtgtggtttgthcchagcatyaaagcyggaaatgacatagcaaactgcytgcgraaaaayggraaaaaggtyatycaacttagtagraaracttttgayacagaatatcaraaracyaractraatgattgggacttygtggtgacdacwgacatttcagaaatgggrgcyaayttyaaagcagayagagtgatygacccaagaagatgtctcaarccagtnatyytgacagahggaccngagcgngtgatyctggcnggaccaatgccagtcaccgyvgcgagygctgcgcaaaggagagggagagttggcaggaacccacaaaargaaaatgaccartacatattcacgggccagccyctyaayaatgaygaagaccaygctcactggacagaagcaaaaatgctgctrgacaacatyaayacaccagaagggathataccagctctctttgarccagaragggagaagtcagccgcyatagacggygartaycgcytgaarggtgagtccaggaagacyttcgtggaactcatgaggaggggwgacctyccagtytggytagtcccataaagtagcntcagaaggratcaaatayacagayagaaaatggtgytttgatggarsarcgyaayaatcaaattttagargagaayatggatgtggaaatctggacaaaggaaggagaaargaaaaaaytgagrcctaggtggcttgaygcccgcacytattcagatcchttagcrctcaargaattcaaggaytttgcrgctggyagraagtcrathgcccttgatcttgtgacagaaataggaagagtgcctwcacayytagcyyayagaacragaaaygchctggayaayytggtgatgytgcayacgtcagaacayggyggbarggcytacaggcatgcrgtggaggaaytaccagaracaatggaaacactcytactchtgggactnatgatcytrttracaggtggrgcdatgcttttcytratatcaggyaaagggattggaaagacttcaataggactcathtgtgtyarthgyttccagygggyatgttrtggatggchganrtyccactycaatggatcgcgtcggcyatagtcctggartttttyatgatggtgytrctnataccagaaccagaaaarcaragaacyccccaagacaaycaactcgcatatgtcgtgataggcatacthacaytggcngcratartagcagcyaatgaaatgggahtrytggaaacyacaaagagagayttrggratgtcyaargarccaggtgttgtytcnccaaccagytatttrgatgtrgahttgcacccagcatcagcctggacattgtaygcygtggccacnacagtaataacaccaatgttragacataccatagagaattcyacagcaaatgtntcyctggcagcyatagcyaaccaggcagtggtcctgatgggtttrgacaaaggatggccdatatcraaaatggacytagghgtrccnctrytggcaytggghtgctattcacaagtgaacccactvactcthacdgcggcagtactyytgctrrtyacrcattatgchatyataggtccaggattgcargchaaagccacycgtgaagctcaraaaagracrgctgctggaataatgaagaatccaacdgtggaygggataatgacaatagacctagatcctgtdatataygattcaaaattygaaaarcaactrggacargtyatgctcctggttytgtgygcagtycaacthttgytaatgagaacatcatgggcdhttgtgtgaagctytaacyctagcyacaggaccaataacaacactctgggaaggatcacctggdaagttytggaacaccacgatagctgtttccatggcnaacathtttagagggagctayttrgcaggagctgggcttgcatttttctathatgaaatcagthggaacaggrarragaggaacrggstcacarggygaaacyttrggagaaaartggaaaaagaarttraatcarttatcccggaaagagtttgacctttaccaagaaatcyggaatcactgaagtrgatagaacagaagccaaagaagggytgaaaagaggagarayaacacaycatgccgtvtccmgagghagctgcaaaactycaatggttygtggaragraacatggtcatyccygaaggaagagtyatagacttrggctgt**ggaagaggaggctggtcatatt**aytgygcaggaytgaaaaaagtyacagaagtgcgaggatacacaacaaggyggyccaggacaygaagaaccacgtaccyatgtcyacatayggatggaacatagtyaagytaatgagtggaaaggahgtgttytatctbccacctgaraagtgtgayachctrttgtgtgacattggagaatcttcaccaagyccaacagtggaagaragcagaacyataagagtyttgaaratggttgarccatggctaaaraayaaccarttttgyattaaagtdttraacccttacatgccaactgtgattgagcacctagaaagaytacaaagraaayatggaggaatgcttgtgagaaayccactytcacgaaactccacgcaygaaatgtactggatatcyaatggyachgghaayatygtckcttcagtcaayatggtdtcyagaytgctactgaacagrttyacvatgacayayagragacccaccathgagaaagatgtggatttrggagcrggracycgacatgthaatgcggaaccagaaacacccaayatggaygtcattggrgaragaataaraaggatcaaggagggagcatadttcaacatggcactatgatgahgaaaayccytayaaaacgtgggcttaccatggatcntatgaagthaargccacaggc**tcagcctcctccatgataaatg**gagtcgtgaaactcctcacnaaaccatgggatgtggtbcccayggtgacacagatggcaatgacrgayacaacycchttyggncagcaragrgthttyaaagagaaagtggacaccaggacdcccarryccatgccaggracaagaarggtnatggrgatyacagcggartggctytggagaaccctgggaaggaayaaaaracccagrytrtgcacragrgargagttyacaaaaaaggtcagaacyaacgcrgcyatggghgchgtnttcyacagaggagaaccaatgggayagygcnaragctgcygttgargaygarganttytggayaacthgtgggacagagaacgtgaactccacaaabtgggcaartgtggaagctgygthtayaayatgatgggcaagagagagaaraaacttggagagtttggcaaagcaaaaggcagtagagcyatatggtacatgtggttgggagccaggtaccttgagttcgargcvcthggattyttaaatgaagaccaytggttctcgcgtgaraactcttacagtggagtrgaaggrgaacggactgcayaagctrggmtayathttragrgayatytcyaagatacchggaggagcyatgtatgcygatgacacagctggntgggacacaagaataacaggaagatgacctgcacaatgaggaaaaratcayacagcaaatggaccctgaacacagrcarytagcdaacgcyatattyaagctcacataccaaaacaaagtggtcaaagtncaacgaccractccaacrrggcacggtaatggacatyatatctagraaagaccaaagrggcagtggacargtrggracttatggnctbaayacatgttcactcaaacatggaagcccagttrrtcagacaratggaaggagaaggygtgytgwcraagrcagacctcgagaaccctcatctgyyagagaagaaaatyacacaatggttggaaachaaaggagtggagagrttaaaaagaatggccathagcggrgatgattgcgtdgtgaaaccratygaygacaggttygcyaatgccctgcthagcyctgaaygayatgggaaargttmggaargacataccycaatggcagccatcraagggntggcatgattggcaacaggtbcctttctgctcccaccactttcatgaaytgatyatgaaagatggaagaaagttggtrgtyccctgcagaccycaggaygaaytaataggragrgcdagaathtctcarggagcrggatggagcctyaragaaachgcatgyctrgggaaagcctacgchcaaatgtggagctctcatgtattttcacagaagagayctyagaytagcatccaacgccatwtgttcagcagtaccagtccaytggrtyccyacaagyagaacgacdtggtcyatycaygctcaccatcagtggatgachacagaagacatgcthacygtytggaacagggtgtggatmgaggahaatccatggatggaagacaaaactccagtyacaacbtgggaaratgttccatayctagggaagagagaagaycaatggtgyggatcactyathggtctcacttccagagcaacctgggcccagaacatacyyacngcaatycaacaggtdagaagcctyataggcratgaagarttyctggactacatgccttcdatgaagagattyaggaaggargaggagtyrgagggagccatttggtaaaaharyaggaagtgraaaaaaagagaggydaactgtcaggccayhttaagccacagtacggaanaagctgtgcwgcctgtgagccccgtccaaggacgttaaaagaagaagtcaggccccaaaagccacggtttgagcaaaccgtgctgcctgtagctccgtcgtggggacgtaaadacctgggaggctgcaavcygtggaagctgtacgcacrgtgtagcagactagyggttagaggagacccctcccatgacacracgcagcagcggggcccgagcactgagggaagctgtacctccttgcaaaggactagaggttagaggagaccccccgcaaayaaaaaacagcatattgacgctgggagagaccagagatcctgctgtctcctcagcttataacgattgccagtgcacagaacgccagaaaatggaatggtgctagwtgatatcaacaggtgtctagt

**b**

wwtggctgcgtragacacacgtagcctaccagtttcttatctgctctactctgcaaagcaagagactyaakaacccatcatggatyctgtgtacgtggayatagacgctgacagcgcctttttgaaggccctgcaacgtgcgtaccccatgtttgaggtggaaccwaggcaggtcacaycraatgaccatgctaatgctagagcgttctcgcatctagcyataaaactaatagagcaggaaattgaycccgactcaaccatcctggatathggyagtgcgccagcaaggaggatgatgtcggacaggaagtaccactgcgtytgcccgatgcgcagygcrgaagatcccgagagactcgcyaattatgcgagaaagctmgcatctgccgcaggaaaagtcctggacagaaacatytctggaaagatcggggacttacaagcrgtratggccgtgccagacamggagacgccvacattytgcttacacacagaygtmtcatgtagacagagagcagacgtcgckatataccaagacgtctatgctgtacaygcacccacgtcgctataycaccaggcgattaaaggrgtccgaktggcgtactgggtwgggttygacacaaccccgttcatgtacaaygcyatggcgggtgcctacccctcatactcgacaaaytgggcrgatgagcaggtactgaaggctaagaacataggattatgttcaacagacctgacggaaggtagacgaggcaarttgtctatyatgagaggraaaaagctaraaccgtgcgaccgtgtgctgttctcagtagggtcaacgctytacccggaaagccgyahgctacttaagagytggcacctrccatcvgtgttccatytaaagggcaarctyagcttcacatgccgctgtgayacagtggtttcgtgygarggctacgtcgtyaagagaataacgatgagcccaggcctttayggaaaaaccayrgggtaygcggtaacccaccacgcagacggattcytghtgtgcaagachaccgayacggttgacggcgaaagagtgtcattctcggtgtgcacrtacgtgccggcdaccatttgtgatcaaatgaccggcatccttgctacagaagtcacgccggaggatgcacagaagctgttggtggggctgaaccagagratagtggttaacggcagaacgcaacggaayaygaacaccatgaaraaytayhtrmttcccgtggtygcccargccttcagtaagtgggcraaggagtgccggaargayatggaagatgaraarctyctgggggtcagagaaagaacactracctgctgctgtctdtgggcattyaagaagcagaaaacacacacggtctacaagaggcctgatacccagtcaatycagaaggttcaggccgartttgacagctttgtrgtaccrrgyctgtggtcgtccgggttgtcaatccckttgaggacyagaatcaartggytgttamgcaaggtgccraaarchgacctgaycccatacagcggdraygcccragaagcccrggaygcagaaaaagaagcagagga**agaacgagaagcagaactgact**cdygargchctaccacchctwcaggcagcacaggaagatgtycaggtcgaaatcgacgtggaacarcttgaggayagagckggbgcwggaataatagagactccgagaggmgctatyaaagttactgcccaacyaacmgaccacgtcgtgggrgagtacytggtwctytccccgcagaccgtaytacgnagccaraagctyagyctgatycacgchttrgcggagcaagtgaagacgtgyacgcayarcggacgagcagggaggtatgcggtcgaagcgtacgaygscmgagtyctagtgccctcaggctaygcaathtcgcctgaagacttccagagyctaagcgaaagcgcracgatggtgtayaacgaaagagagttcgtaaayagaaagytacaccayattgcgatgcayggaccagccctgaacacygacgaagagtcgtatgagctkgtgagggcagagagracagaacaygagtacgtctacgaygtggaycaragaagatgctgtaagaaggaagaagchgcaggaytggtactggtgggcgacttgactaatccgccctaccacgaattygcataygaagggctaaaaatycgcccygcytgyccatacaaaaytgcagtcataggagtcttcggrgtaccrggatctggyaagtcagchattatcaagaacctagttaccaggcargacctggtgactagcggaaagaaagaaaactgccaagaaatcascaccgacgtgatgagacagagaggtctagaratatctgcacgtacggthgaytcgctgctcttgaatggatgyaayagaccagtcgacgtgttgtacgtagacgaggcgtttgcgtgccactctggaacgytacttgchttgatcgccttggtgagaccaagrcwgaaagttgtactdtgtggtgacccgaagcagtgcggcttcttcaatatgatgcagatgaaagtcaactayaatcayaacatctgcacycaagtgtaccacaaaagtatctccaggcggtgtacactgcctgtgacygccattgtgtcvtcgttgcattacgaaggc**aaaatgcgcactacgaatgagt**acaacawgccgattgtagtggacactacrggctcaacraaacctgaccctggagayctcgtgttaacgtgcttcagaggrtgggttaaacaaytgcaaattgactatcgtggayacgaggtcatgacagcagccgcatcccaagggttaacyagaaaaggagtttacgcagttagrcaaaaagttaaygaaaacccvctytatgcatcaacrtcagagcacgtcaacgtactcctaacgcgtacggaaggtaaactggtatggaagacactytcygghgacccgtggataaagacgctgcagaacccaccgaaaggaaacttcaargcaactattaaggagtgggaggtggagcaygcatcrataatggcgggcatctgcagtcaccaartgaccttygayacrttccaaaayaaagccaacgtttgytgggctaagagyttggtccctrtcctcgaaacagcggggataaaactaaaygayaggcagtggtcycagataattcaagccttcaaagaagacaaagcataytcaccygaagtagccctgaatgaaatatgyacgcgcatgtatggggtggatctagacagygggctattytctaaaccgttggtrtctgtgtattacgcggataaccaytgggataayaggcckggaggraaratgttcggattyaacccygaggcagcrtccattctagaaagaaagtayccrttyacaaaaggraagtggaacatcaacaagcagatctgcgtgacyaccaggaggatagaagacttyaaccctaccaccaacatyataccggycaacaggagactaccacactcattagtggccgaacaccgcccagtaaaaggggaaagaatggaatggytggttaacaagataaacggmcaycaygtrctcctggtyagyggctrtarccttgcactgcctactaagagagtcacytgggtagcgccrytaggygtccgcggagcggactayacatacaacytagagytgggtctrccrgcaacrcttggtaggtatgacctwgtggtcataaacatccacacaccttttcgcatacaccattaycaacagtgcgtvgaycacgcaatgaaactgcaaatgctmgggggtgactcaytgagactgctcaaaccgggyggctctctattgatcagagcatayggttacgcagatagaaccagtgaacgagtmatctgcgtaytgggacgyaagtttagatcrtcyagagcrttgaaaccaccdtgtrtcaccagyaayactgagatgtttttyctattyagcarytttgayaatggcagaaggaatttyacaackcatgtcatgaacaatcaactgaaygcagccttygtaggacaggycacccgagcaggatgtgcaccrtcgtaccgggtaaaacgcatggayatcgcgaagaacgatgaagagtgcgtrgtyaacgccgcyaaccctcgcgggttaccrggtgacggygtttgcaaggcagtatayaaaaartggccggagtcctttaaraayagtgcaacaccagtrggaacygcaaaaacagtyatgtgcggtacrtatccagtaatccacgchgtwggaccaaacttctcwaattaywcggagtcygaaggggaccgrgaattggcrgctgcctatcgagaagtcgcaaaggargtaactagrctgggagtaaatagygtagctatacctctcctctccacaggtgtatactcaggagggaaagacaggctracccagtcactgaaccacctctttacagccatggactcgacggatgcagacgtggtcatctactgccgmgacaargaatgggagaagaaaatatctgaggccatacagatgcggacccaagtrgagctgctggatgagcacatctccatagaytgcgatrtyrttcgcgtgcaccctgacagyagyttggcaggyagaaaaggatacagcaccacggaaggcgcactgtaytcatatctagaagggacmcgttttcaycaracggcwgtggatrtggcrgagatayayactatgtggccaaagcaaayagaggccaaygagcaagtytgcctatatgccctgggggaaagtattgaatcratcaggcagaaatgcccggtggatgatgcagaygcatcatctcccccvaaaactgtcccgtgyctytgccgktaygcyatgacwcchgaacgcgtyacccgrcttcgcatgaaycaygtcacaaryataattgtgtgttcttcrtttcccctyccaaagtacaaratagaaggagtgcaaaaagtcaaatgctcyaaggtaatgytattygaycacaaygtgccatcgcgcgtaagtccaagggaatayadaycttcccaggartctgyacrggargygagtayrrymacgtcaytgacgcatagycarttygayctaagcgyygayggcragahactgccygtcccgycagacctggatgctgacgccccrgcccyagaaccrgcmctwgacgacggggcgryacayacdytrccahcyryaayyggaaaccttgcggccgtgtctgaytgggtaatgaryaccgyrccwgtcgcrccrccyagaagaagrmgwgggaraaacytgamygtsaymtgygaygagagagaagggaayrtamywcccatggctagcgtycgrttcttyagrgcdgakcwryryycvrycgyacargaaacrgcrgagayrcgygayacrgchryktcyctycrggcrccvcyragtrycrcyryrgaacygarycahcyrccgatctcmttyggwgcaycaarygagackttccccathacrttyggggayttyraygawggrgaratygaaagcttgtcytytgagytrctracyttyggrgacttcyyrccvgghgaagtggatgayytgacagayagcgactggtccacgtgyycagacacggacgacgarttaygact**agacagggcaggtgggtatata**ttctcgtcdgacacyggtccaggycatttacaacagaagtcdgtacgccagtcagtgctgccggtraacaccytggaggaagtycacgaggagaagtgttacccacctaagctggatgaakyaaaggagcaactaytacttaagaaactccaggaragtgcrtccatggccaayagaagcaggtatcartcvcgcaaagtrgaaaayatgaaagcarcaatcatycagagaytaaagagaggctgtaraytdtayttaatgkcagagaccccraaagtccckacytaycggacyryatayccggcgcctgtgtactcgcctccgatyaaygtccgaytgtccaaycccgagtchgcagtggcagcvtgyaatgagttcytrgctagaaactayccaactgtytcatcataccaaatyaccgaygagtaygatgcatatctagacatggtggacgggtcggagagttgyytggaccgrgcgacrttcaatccgtcaaaactyaggagctacccraaacarcaygcttaycacgcgccytchatcagaagcgctgtaccktccccattccagaacacactacagaatgtactggcagcagccacgaaaagraactgcaacgtcacacagatgagggaaytacccactttggactcrgcagtattcaacgtggagtgtttyaaaaaattygcatgyaaccragaatactgggaagaatttgchgccagccctatyaggataacaactgagaatytarcaacctatgtyactaaaytaaargggccaaaagcagcagcgytrttygcaaraacccataatctrctgccrctrcaggadgtrccaatggataggttcacagtagayatgaaaagggaygtraaggtractcctggtacaaagcayacagaggaaagrcctaargtrcaggttatacaggcrgctgamcccttggcracagcrtacctatgtggrattcacagagarytggttaggagrytgaacgccgtyctcctacccaatgtrcatacactatttgacatgtctgccgaggayttcgatgccatyatagccgcacacttyaagccaggagacrcygttttrgaaacggacatagcctcctttgataagagccargatgaytcacttgcgcttacygcyttratgctgttagargatttrggrgtggatcactccctgytggacytgatagaggctgctttyggagagatttccagctgtcayctrccgacaggtacgcgcttcaagttcggcgcyatgatgaaatchggtatgttcctaactctgttcgtcaacacrytgytaaayatcaccatcgcyagccgrgtgytggaagatcgtctgacaaaatcygcrtgcgcrgccttcatcggcgacgacaacataatacatggdgtcgtctccgatgaabtgatggcagccagatgygcyacttggatga**acatggaagtgaagatcataga**tgcagttgtatccydgaaagcyccttacttttgtggagggtttatactgcaygatayhgtgacaggaacagcttgcagagtggcrgacccgctaaaaaggytdttyaaaytgggcaaaccgytagcggcaggtgacgaacaagaygavgayagaagacgdgcgctggcygaygaagtrrtcagatggcaacgaacagggctaatwgatgagytggagaaagcggtryactcyaggtaygaagtgcagggtatatcagttgykgtaatgtcyatggccacctttgcaagctccagatcyaacttcgagaagctcagaggacccgtcrtaactttgtacggcggtcctaaataggtacgcactacagctacctattttgtcagaagccgacagyargtayctaaayacyaatcagcyayaatggagttyatcccaacccaaactttytacaayaggaggtaccagcctcgaccytggactccgcgcyctactatccaarthatyagrcccagaccgcgyccdcaraggmargcygggcaacttgcccagctgatctcagcagttaataaaytgacaatgcgcgcggtaccycaacagaagccdcgcargaatcggaagaataagaagcaaaagcaaaarcarcaggcgccacraaacrayayraabcaaaagaagcagccvcctaaaaagaaaccrgytcaaaagaaaaagaagccgggccgyagagagagratgtgcatgaaaatcgaaaatgaytgyatyttcgaagtcaagcaygaaggtaaggtaacaggttacgcgtgcytggtrggggacaaagtaatgaarccagcacaygtaaaggggaccatcgataaygcggacctggccaaaytggccttyaagcggtcatctaagtaygaccttgaatgcgcgcagatacccgtgcacatgaagtccgacgcttcgaagttcacccatgagaaaccggaggggtactayaactggcaccacggagcagtacagtactcaggaggccgrttcaccatcccbacaggtgcdggcaaaccaggggayagyggyagaccgatcttcgacaacaagggrcgcgtggtggccatagtyttaggaggagctaatgaaggagcccgtacagccctctcvgtggtgacctggaayaaagacatygtcacdaaaatcaccccygagggrgccgaagagtggagtctbgccatyccagttatgtgcctgytggcaaayaccacgttcccctgctcccagcccccttgcryrccctgctgctacgaaaargarccggagraaaccytrcgcatgctwgargacaaygtcatgagmccygggtactatcagctgctacaagcatcvttaacatgttctccccrmcgccrgcgrcgcagyayyaaggacmacttcaatgtctataaagccayaagaccrtacytagctcactgtcccgactgtggagaagggcactcgtgccatagtcccgtagcrctagaacgcatcagaaaygaagcgacagacgggacgytgaaaatccaggtytccttgcaaatyggaatarrgacggatgayagccaygattggaccaagctgcgttayatggayaaycayatrccagcagacgcagrgmgggcbgggctryttgtaagaacdtcagcaccrtgyacgattactggracaatgggacacttcatyctggcccgwtgtccraaaggrgaaactctgacggtgggrttcactgayrgtagraagatyagtcaytcatgtacgcacccatttcaccacgaccctcctgtgataggycgggaaaaattycattcccgaccgcagcacggtarrgarytaccttgcagcacgtacgygcagagyamcgcygcaactrccgaggagatagaggtacayatgcccccagacaccccwgatcgcacattrmtgtcacaacagtccggyaaygtaaagatcacagtyaatrgbcagacggtgcggtayaagtgyaattgyggtgrctcaartgaaggaytaaymactacagayaaagtgattaataactgcaaggttgatcaatgycatgccgcggtcaccaatcacaaaaadtggcagtataaytcccctctggtcccgcgyaaygytgaayyyggggaccgdaaaggaaaarttcacatyccrtttccdctggcaaatgtracatgcagggtgcctaargcaagraaccccaccgtgacgtacggraaaaaccaagtcaycatgytrctgtatcctgaccacccaacrctcctgtcctacmggartatgggagaa**gaaccaaactatcaagaagagt**gggtgaygcayaagaaggarrtcrbgytaaccgtgccgactgargggctcgaggtyacgtggggyaacaaygagccgtayaagtattggccgcaghtatcyrcaaacggtacagcccayggccacccgcatgagataatyytgtaytattatgagctgtaccchactatgactgtrgtagttgtgtcagtggccwcgttcrtactcctgtcgatggtgggtrtggcagygggratgtgcatgtgtgcacgacgcagatgcatyacaccrtaygaactgacaccaggagctaccgtccctttcctgctyagcctaatatgctgcatyagaacagctaaagcggccacataccavgaggcygcgryatacctgtggaacgagcagcarcctttrttttggctrcaagcccttattccgctggcagccctgattgtyctatgyaactgtctgagactcttaccatgytkytgtaaaaygttgrcttttttagccgtamtgagcrtcggtgcccacactgtgagcgcgtacgaacacgtaacagtgatcccgaacacggtgggagtaccgtataagactctagtcaayagacckggctacagccccatggtaytggagatggarctwctgtcwgtcactttggagccaacdctatcgcttgattacatcacgtgcgartayaaaaccgtyatcccgtctccgtacgtgaartgctgcggtacagcagagtgyaaggacaararcctacctgaytacagctgtaaggtcttcaccggcgtctacccattyatgtggggcggcgcctactgcttctgcgacrcygaaaayacgcarttgagcgaagcacaygtgg**agaagtccgaatcatgcaaaac**agaatttgcatcagcatayagggctcataccgcatcbgcatcagctaagctccgcgtcctttaccaaggaaataayrtyactgtadctgcytatgcaaacggcgaycatgccgtcacagthaaggacgcyaaattcathgtggggccaatgtcttcagcctggacacctttygacaayaaaatygtggtgtacaaaggygacgtytayaacatggactacccgccyttyggcgcaggaagaccaggacaatttggcgayatccaaagtcgcacrcctgaragyraagacdtctatgctaayacacaactggtactgcagagaccgkchgygggtacggtrcaygtgccrtactctcaggcaccatctggcttyaagtattggytaaaagaacgmggggcgtcrctrcagcacacagcaccatttggctgycaaatagcaacaaacccggtaagagcgrtraactgcgccgtagggaacatgcchatctccatcgacataccggahgcggccttyacyagggtcgtcgacgcgccmtctttaacggacatgtcrtgygaggtaycagcctgcacccaytcctcagactttgggggcgymgccatyattaaatatgcagycagyaagaaaggcaagtgtgcrgtgcaytcgatgacyaacgccgtcactatycgggaagctgavatagaagtdgaagggaaytctcagytgcaaatctctttytygacggccytrgccagcgccgaattccgcgtacaagtctgttctacacaagtacactgtgcagcygagtgccaycchccgaargaccayatagtcaaytacccggcgtcacayaccacccycggggtccargacathtccgytacggcgatgtcatgggtgcagaagatcacgggaggtgtgggactggttgtygctgttgchgcactgatyctaatcgtggtgctatgcgtgtcgttyagcaggcactaacttgacraytargyaygaaataactaaatagcaaaagtagaaagtacataaccargyataygygtcccywaagagacacacyryayatagcwaakaatcaatagataagtdtagatcaaagggctrhryaayccctgaatagtaacaaaatayaaaaatcamyaaaaaatyataaaawaraaaamharaaaamanaraadtabrtaarhaggtatahgtgtcccctaagagacacayyrtatrtagbtaagaatcaatagataagyatagatcaaagggcygaaytaacccctgaatartaacaaaatatraaaayyaataaaaaatcataaaatagaaaaaccataaacagaagtagttyaaagggctatraaaacccctgaatagtaacaaaayataaarygtaataaaaatyaaaygtgtacccaaaagaggtacagtaagaatcagtgaataccataattggcaahcggaagagatgtaggtacttaagcttcctaaaagcagccgaactcvctttgagahgtaggcrtagcabaccgaactcttccaydattctccgaacccacagggacgtaggagawgttmwwwkkkdytwwawwwwyyyhraacagtaataaaacataaaattaataaggatcaaatgagtaccataattggcaaacggaagagaygtaggtacttaagcttcctaaaagcagccgaactcactttgagatgtaggcatagcataccgaactcttccacaattctccgtacccatagggacgtaggagatgttattttgtttttaatatttc
