# Supplementary material for: Synthetic miRNAs induce dual arboviral-resistance phenotypes in the vector mosquito Aedes aegypti
Source: Commun Biol. 2018 Feb 8;1:11. doi: 10.1038/s42003-017-0011-5 (PMC6053081; doi:10.1038/s42003-017-0011-5)
Supplement: Supplementary file 3 — Description of Additional Supplementary Files [file 42003_2017_11_MOESM3_ESM.docx]

**Description of Additional Supplementary Files**

File Name: Supplementary Data 1

Description: **Consensus sequences**. **a** DENV-3. **b** CHIKV. Consensus sequences based on 356 strains of DENV-3 and 32 strains of CHIKV. Bold and underlined regions are the binding sites of antiviral miRNAs.
